# Environmental risk factors of inflammatory bowel disease: toward a strategy of preventative health

**DOI:** 10.1093/ecco-jcc/jjaf042

**Published:** 2025-03-11

**Authors:** Tarun Chhibba, Beatriz Gros, James A King, Joseph W Windsor, Julia Gorospe, Haim Leibovitzh, Mingyue Xue, Williams Turpin, Kenneth Croitoru, Ashwin N Ananthakrishnan, Richard B Gearry, Gilaad G Kaplan

**Affiliations:** Division of Gastroenterology & Hepatology, Temerty Faculty of Medicine, University of Toronto, Toronto, Ontario, Canada; Department of Gastroenterology and Hepatology, Reina Sofía University Hospital, IMIBIC, University of Córdoba, Córdoba, Spain; Liver and Digestive Diseases Networking Biomedical Research Centre (CIBEREHD), Madrid, Spain; Departments of Medicine and Community Health Sciences, University of Calgary, Calgary, Alberta, Canada; Departments of Medicine and Community Health Sciences, University of Calgary, Calgary, Alberta, Canada; Departments of Medicine and Community Health Sciences, University of Calgary, Calgary, Alberta, Canada; Division of Gastroenterology & Hepatology, Temerty Faculty of Medicine, University of Toronto, Toronto, Ontario, Canada; Zane Cohen Centre for Digestive Diseases, Lunenfeld Tanenbaum Research Institute, Mount Sinai Hospital, Toronto, Ontario, Canada; Division of Gastroenterology & Hepatology, Temerty Faculty of Medicine, University of Toronto, Toronto, Ontario, Canada; Zane Cohen Centre for Digestive Diseases, Lunenfeld Tanenbaum Research Institute, Mount Sinai Hospital, Toronto, Ontario, Canada; Division of Gastroenterology & Hepatology, Temerty Faculty of Medicine, University of Toronto, Toronto, Ontario, Canada; Zane Cohen Centre for Digestive Diseases, Lunenfeld Tanenbaum Research Institute, Mount Sinai Hospital, Toronto, Ontario, Canada; Division of Gastroenterology & Hepatology, Temerty Faculty of Medicine, University of Toronto, Toronto, Ontario, Canada; Zane Cohen Centre for Digestive Diseases, Lunenfeld Tanenbaum Research Institute, Mount Sinai Hospital, Toronto, Ontario, Canada; Division of Gastroenterology, Massachusetts General Hospital and Harvard Medical School, Boston, USA; Department of Medicine, University of Otago, Christchurch, New Zealand; Departments of Medicine and Community Health Sciences, University of Calgary, Calgary, Alberta, Canada

**Keywords:** Crohn’s disease, ulcerative colitis, environmental health, incidence, predisease cohorts

## Abstract

The pathogenesis of inflammatory bowel disease (IBD) involves a complex interplay between genetic, environmental, and microbial factors. Many of these environmental determinants are modifiable, offering opportunities to prevent disease or delay its onset. Advances in the study of preclinical IBD cohorts offer the potential to identify biomarkers that predict individuals at high risk of developing IBD, enabling targeted environmental interventions aimed at reducing IBD incidence. This review summarizes findings from 79 meta-analyses on modifiable environmental factors associated with the development of IBD. Identified risk factors include smoking, Western diets, ultra-processed foods, and early life antibiotic use, while protective factors include breastfeeding, Mediterranean diets rich in fiber, plant-based foods, and fish, along with an active physical lifestyle. Despite the promise shown by observational data, interventional or randomized controlled studies evaluating the efficacy of modifying environmental risk factors remain limited and mostly focus on dietary intervention. This review aims to inform the design of higher quality interventional and randomized controlled studies for disease prevention while providing actionable guidance to healthcare providers on reducing the risk of developing IBD through environmental modifications.

## 1 . Introduction

Inflammatory bowel disease (IBD), which includes Crohn’s disease (CD) and ulcerative colitis (UC), is an immune-mediated condition affecting the bowel.^1^ Although the precise cause of IBD remains unknown, its pathogenesis is thought to result from a complex interplay of genetic susceptibility, alterations in gut microbiota, and environmental exposures.^1^ At the turn of the 21st century, IBD has emerged as a global issue, with prevalence exceeding 0.5% in early industrialized nations of North America, Europe, and Oceania. Additionally, the incidence is rapidly increasing in newly industrialized regions, including Asia, Africa, and Latin America.^2–4^ By 2030, the prevalence of IBD in several early industrialized countries is projected to reach 1% of the population.^5,6^

The role of genetics in IBD is well-established, as demonstrated by family and twin studies and further corroborated by the discovery of the first susceptibility gene, NOD2.^7^ Genome-wide-association studies have identified over 200 loci associated with CD and/or UC.^8^ Fine-mapping with high-density genotyping has pinpointed 45 causal variants.^9^ Despite these advances, genetic heritability accounts for only a fraction of IBD cases.^10^

The importance of environmental factors in IBD pathogenesis is underscored by several epidemiologic patterns: The incidence of IBD increased dramatically in the early industrialized world during the 20th century^2^; immigrants to high-prevalence regions exhibit an elevated risk of developing IBD^11^; and the incidence is rising in newly industrialized countries undergoing westernization.^4,12^

**Figure 1.**
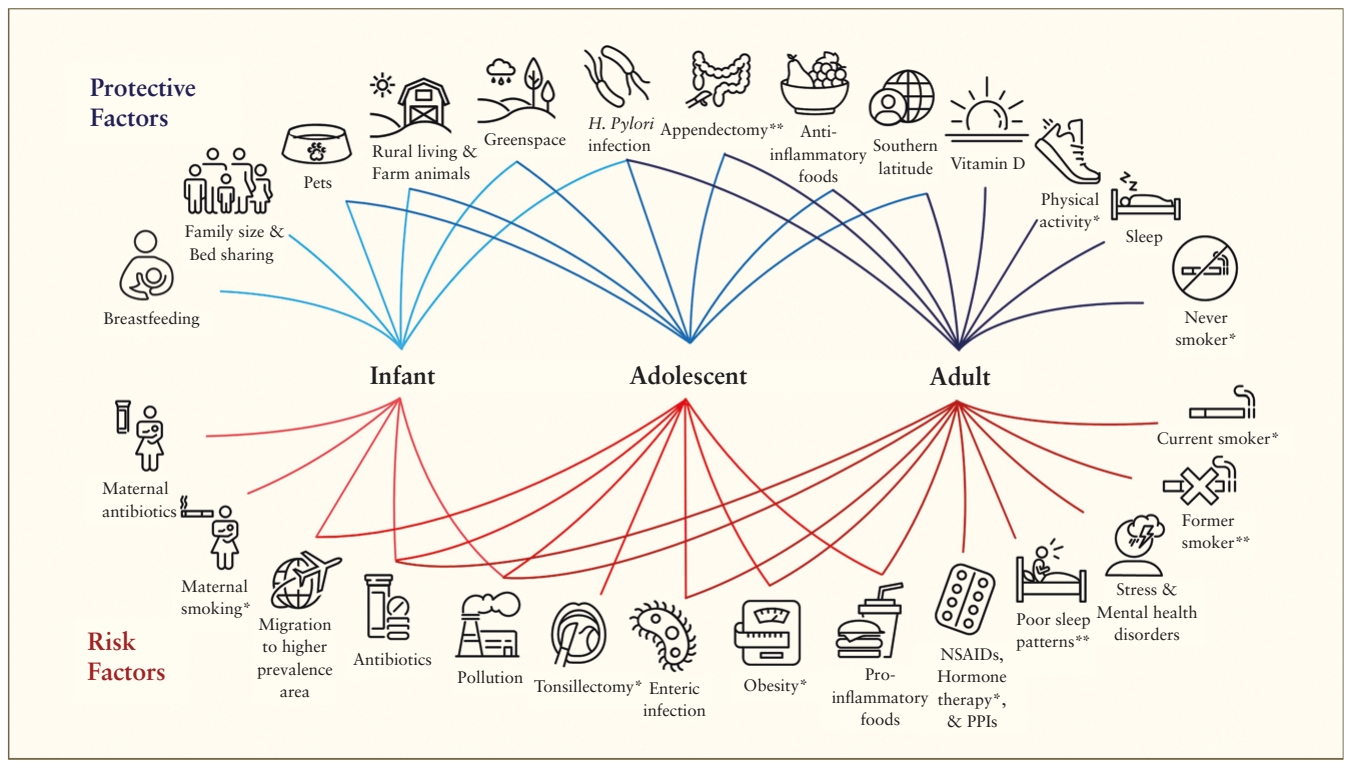
Environmental determinates of Crohn’s disease and ulcerative colitis across the age continuum. “*” are environmental factors associated exclusively with Crohn’s disease, while “**” indicates factors associated exclusively with ulcerative colitis.

Inflammatory bowel disease can be diagnosed at any age, though the most common age of diagnosis is during adolescence and early adulthood.^13^ Environmental exposures that influence IBD development have varying effects depending on the age at exposure (Figure 1).^14^ Early life exposures such as breastfeeding, antibiotic use in the first year of life, exposure to livestock and pets, the number of siblings, and population density are believed to modulate the risk of developing IBD—possibly through their effects on the development of the intestinal microbiome (Supplemental Table).^15,16^ Later in life, lifestyle-related environmental factors, such as diet and smoking, begin to exert a more significant influence on the onset of IBD in adulthood (Figure 1).^15^

To address the rising prevalence of IBD, it is imperative to reduce the incidence of IBD.^17,18^ Achieving effective disease prevention requires a deeper understanding of the underlying mechanisms through which environmental factors influence disease development. In some cases, environmental determinants may be modifiable, offering potential targets for interventions to lower the likelihood of disease onset.

In the first part of this review, we summarize current knowledge on modifiable environmental determinants of IBD by synthesizing findings from meta-analyses of environmental risk factors associated with its development. Subsequently, we interpret these data to propose future strategies at both individual and population levels for reducing IBD risk. Our objective is to provide a roadmap for translating these findings into actionable strategies to reduce disease incidence.

## 2. Methods

We conducted a PubMed search on November 30, 2024, to identify English-language meta-analyses evaluating modifiable environmental risk factors associated with the development of IBD. Two investigators (B.G., T.C.) independently reviewed titles and abstracts in duplicate and performed a full-text review of selected manuscripts. Any discrepancies were resolved through consensus. Environmental factors were classified as modifiable if they could be influenced or altered by individuals at risk or by broader population-level interventions. We included risk factors supported by at least one meta-analysis related to the factor of interest. Given the heterogeneity in study designs, populations, and time frames, risk factors with at least one associated meta-analysis were considered to have sufficient evidence for inclusion in this review. Our search identified a total of 79 meta-analyses (Supplemental File: Search Strategy, Supplemental Table).

## 3. Potentially modifiable environmental risk factors of IBD

### 3.1 Hygiene and the microbiome

The gastrointestinal tract hosts a complex and diverse community of microorganisms, collectively known as the gut microbiome. The development of the gut microbiome begins early in life, possibly even in utero.^19^ A properly functioning gut microbiome is crucial for appropriate immune function, carbohydrate and bile salt metabolism, synthesis of essential vitamins, and inhibition of pathogens such as *Clostridioides difficile*.^20^ Dysbiosis of the intestinal microbiome, which involves an imbalance in the composition and diversity of these microorganisms, has been implicated in the pathogenesis of both UC and CD.^21^ Emerging research also suggests that dysbiosis is not limited to bacterial species, with the virome and mycobiome increasingly recognized as potential contributors to IBD.^22,23^

The Hygiene Hypothesis offers a potential explanation for the development of chronic immune-mediated diseases, including IBD.^24^ According to this hypothesis, children raised in relatively sterile, urban environments lack exposure to a diverse range of microorganisms. As a result, their immune systems may later overreact to microbes, leading to a dysregulated immune response and, ultimately, the development of IBD.^24^ This hypothesis is supported by observations that early life protective factors—such as large family size, household crowding, younger birth rank, drinking unpasteurized milk, living on a farm, and raising pets—are associated with a reduced risk of IBD.^25^

Since the gut microbiome is largely established within the first 2 years of life, it is plausible that the foundation of the Hygiene Hypothesis lies in early life exposures that influence the microbiome toward dysbiosis.^26^

### 3.2 Infections

Although the exact mechanism underlying the Hygiene Hypothesis remains unclear, alterations in the gut microbiome caused by gastrointestinal infections may contribute to the development of IBD by promoting dysbiosis. A meta-analysis of 10 studies demonstrated that Asians with IBD had a significantly reduced proportion of *Helicobacter pylori* infection vs controls (relative risk (RR): 0.48; 95% CI, 0.43-0.54).^27^ Another meta-analysis of 39 studies confirmed a similar trend across various age groups and ethnicities, showing that *H. pylori* infection was associated with a lower risk of IBD (odds ratio (OR): 0.43; 95% CI, 0.36-0.50).^28^ Further analysis into different genotypes of *H. pylori* revealed that individuals with *H. pylori* carrying the Cytotoxin-associated gene A (CagA) were less likely to develop IBD compared to those without *H. pylori* exposure (OR: 0.23; 95% CI, 0.15-0.35). However, those who were seronegative for *H. pylori* CagA did not show a decreased risk of developing IBD compared to individuals without *H. pylori* (OR: 0.74; 95% CI, 0.46-1.17).^29^

Numerous studies have also investigated the risk of IBD following an episode of acute gastroenteritis, finding an increased risk of developing IBD within the first year after infection. A cohort study of over 13 000 individuals with IBD from 1991 to 2003 demonstrated an increased risk of IBD following infections with *Campylobacter* and *Salmonella* (OR: 2.9; 95% CI, 2.2-3.9).^30^ A case-control study of 20 790 individuals with gastroenteritis demonstrated a higher risk of subsequent IBD in those with a prior bacterial (aOR: 2.02; 95% CI, 1.82-2.24), parasitic (adjusted odds ratio (aOR): 1.55; 95% CI, 1.03-2.33), or viral (aOR: 1.55; 95% CI, 1.34-1.79) infection.^31^

### 3.3 Early life exposures

Several perinatal risk factors have been identified as influential in the development of IBD. One such factor is the use of perinatal antibiotics, which are commonly prescribed during pregnancy and have been shown to cause alterations in the infant microbiome.^32^ However, studies indicate that the rate of antibiotic prescriptions for maternal infections is similar between mothers of individuals with IBD and mothers of controls (OR: 0.86; 95% CI, 0.68-1.09). Despite this, a large population-based study of 827 239 children found that exposure to antibiotics during pregnancy was associated with an increased risk of IBD in offspring (HR: 1.93; 95% CI, 1.06-3.50). The third trimester appeared to be the most critical period, as analyses revealed that only antibiotic exposure during this time was significantly associated with an increased risk of IBD.^33^ Similarly, a population-based cohort study from Denmark reported an elevated risk of IBD development with exposure to 3 or more courses of antibiotics during pregnancy (hazard ratio (HR): 1.29; 95% CI, 1.03-1.62).^34^ A meta-analysis of 4 observational studies further supported this finding, showing that antibiotic use during pregnancy was associated with an increased risk of IBD in offspring later in life (OR: 1.75; 95% CI, 1.22-2.51).^35^

A meta-analysis of early life exposures determined that maternal smoking during pregnancy increased the risk of IBD in offspring (OR: 1.49; 95% CI, 1.17-1.90), while maternal age did not appear to have a significant effect (OR: 0.86; 95% CI, 0.45-1.65).^35^ The 2 most recent meta-analyses concluded that C-section (OR: 1.00; 95% CI, 0.75-1.33)^36^ and mode of delivery (OR: 1.01; 95% CI, 0.81-1.27)^37^ were not significantly associated with the risk of IBD.

### 3.4 Breastfeeding

Breast milk may reduce the risk of developing IBD through its bioactive components, including immunoglobulins, cytokines, and human milk oligosaccharides, as well as the transfer of maternal microbiota and immunoglobulins to the infant. These components promote gut microbiota diversity, strengthen the intestinal barrier, and modulate immune responses, thereby reducing inflammation.^38^ The Environmental Determinants of Diabetes in the Young study demonstrated breastfeeding as the most significant factor in shaping the gut microbiome in early life.^38^ A meta-analysis comparing the gut microbiomes of exclusively breastfed infants with those who were not breastfed showed that differences in gut microbiota persist beyond 6 months of age.^39^ One meta-analysis demonstrated ORs of 0.62 (95% CI, 0.67-0.86) for UC and 0.77 (95% CI, 0.61-0.96) for CD in relation to breastfeeding.^40^ Another meta-analysis confirmed a significant negative association between breastfeeding and IBD overall (OR: 0.74; 95% CI, 0.66-0.83).^41^ The duration of breastfeeding also appears to influence the risk of developing IBD: Breastfeeding for a longer duration—12 months compared to 3 months—was associated with a lower likelihood of developing CD (OR: 0.20; 95% CI, 0.08-0.50) and UC (OR: 0.21; 95% CI, 0.10-0.43).^41^

### 3.5 Diet

**Figure 2.**
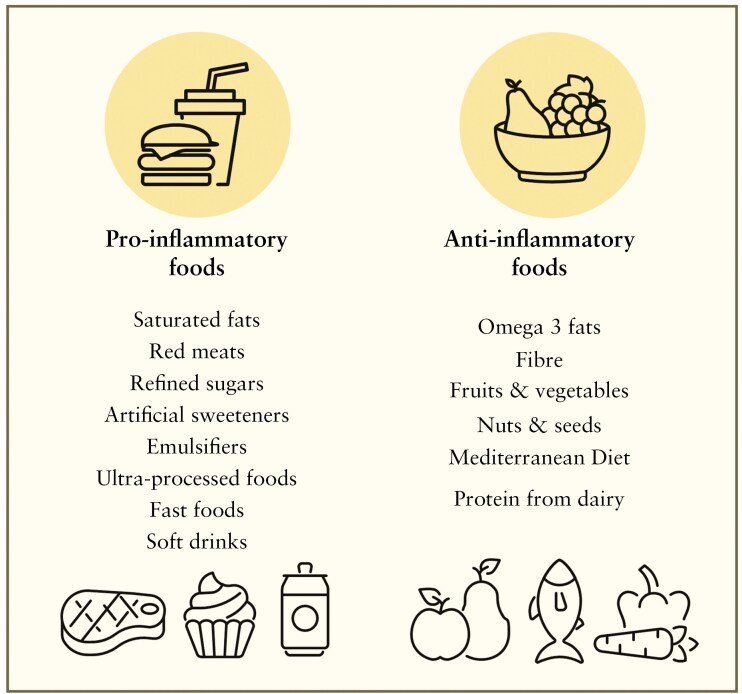
Dietary stratification of pro-inflammatory and anti-inflammatory foods.

The incidence of IBD has risen in newly industrialized countries, coinciding with the adoption of westernized diets.^42^ Diet can alter the characteristics and modify the diversity of intestinal microbiota, which may contribute to the onset of IBD.^43^ Extensive research has explored the impact of individual food groups, macronutrients, overall diet composition, and food processing on the microbiome and their roles in modulating the risk of IBD, with the aim of identifying pro- and anti-inflammatory dietary recommendations (Figure 2).^42^

A systematic review of 19 studies examining the effect of macronutrients on IBD development concluded that high intake of saturated fats, monounsaturated fatty acids, total polyunsaturated fatty acids (PUFAs), total omega-3 fatty acids, and omega-6 fatty acids was associated with an increased risk of CD.^44^ High intake of total fats, total PUFAs, and omega-6 fatty acids were associated with an increased risk of UC. A separate systematic review and meta-analysis indicated that the source of protein influences the risk of IBD. A dose-dependent correlation was observed between meat intake and IBD risk, with each 100-gram per day increase in meat consumption raising the risk by 38%. In contrast, protein from dairy sources was found to be negatively associated with IBD (RR: 0.81; 95% CI, 0.72-0.90).^45^ Meat consumption has also been shown to increase the risk of both UC and CD across Eastern and Western populations.^46^

Fiber intake was associated with a reduced risk of CD (RR: 0.59; 95% CI, 0.46-0.74), but not UC (RR: 1.09; 95% CI, 0.88-1.34).^47^ Conversely, fruit and vegetable consumption has been associated with a lower risk of developing IBD. In a population-based cohort study, high fruit intake was associated with a lower risk of UC development (OR: 0.59; 95% CI, 0.4-0.88).^48^ A meta-analysis confirmed that eating fruit was associated with a lower risk of UC (RR: 0.69; 95% CI, 0.55-0.86) and CD (RR: 0.47; 95%: 0.38, 0.58).^47^ Similarly, vegetable intake was associated with a lower risk of both CD (RR: 0.52; 95% CI, 0.46-0.59) and UC (RR: 0.56; 95% CI, 0.48-0.66).^47^

Western diet has been identified as a risk factor for IBD (RR: 1.92; 95% CI, 1.37-2.68),^49^ whereas the Mediterranean diet has been strongly associated with a reduction in subclinical inflammation, with nearly 50% of this anti-inflammatory effect being mediated by the microbiome.^50^ Fast food and ultra-processed foods also appear to be significant risk factors for IBD. A population-based cohort study found that frequent fast food consumption was associated with developing CD (OR: 2.26; 95% CI, 1.76-4.33) and UC (OR: 2.91; 95% CI, 1.54-5.58).^48^ Meta-analyses have shown that soft drink consumption is associated with an increased risk of developing CD (RR: 1.43; 95% CI, 1.01-1.98)^51^ and UC (RR: 1.69; 95% CI, 1.34-2.30).^52^ Specifically, sucrose intake was associated with a higher risk of CD (RR: 1.09; 95% CI, 1.02-1.16)^53^ and UC (RR: 1.10; 95% CI, 1.02-1.18).^54^

Additionally, highly processed foods have been associated with an increased risk of IBD in both the Nurses Health Study (NHS) and the Prospective Urban Rural Epidemiology cohort studies.^55,56^ A meta-analysis further confirmed that ultra-processed food intake was associated with a higher risk of CD (HR: 1.71; 95% CI, 1.37-2.14), though not UC (HR: 1.17; 95% CI, 0.86-1.61).^57^

### 3.6 Smoking

Current smokers have nearly a 2-fold increased risk of developing CD.^58^ In contrast, former smokers are associated with a higher risk of UC compared to never smokers or current smokers. Smoking has been shown to diminish intestinal permeability, alter microcirculation and cytokine production, and increase mucin production.^59^

Ethnicity may influence the impact of smoking on IBD. Smoking was strongly associated with an increased risk of CD in White populations (RR: 1.95; 95% CI, 1.69-2.24), a pattern not observed among Asian, Jewish, or Latin American ethnicities (RR: 0.97; 95% CI, 0.83-1.13).^60^ Exposure to secondhand smoke does not appear to increase the risk of IBD. A meta-analysis found no significant relationship between childhood secondhand smoke exposure and the development of IBD.^61^

### 3.7 Physical activity

Regular physical activity may reduce the risk of IBD by decreasing systemic inflammation, enhancing gut microbiota diversity, and improving immune regulation. It promotes the production of anti-inflammatory cytokines and helps mitigate stress.^62^ A meta-analysis of 6 studies found that high levels of physical activity were associated with a reduced risk of developing CD (RR: 0.63; 95% CI, 0.50-0.79) and showed a near-significant reduction in the risk of UC (RR: 0.82; 95% CI, 0.68-1.00).^63^ A 2024 meta-analysis confirmed that physical activity was linked to a lower likelihood of developing CD in both cohort (RR: 0.78; 95% CI, 0.68-0.88) and case-control (RR 0.87; 95% CI, 0.79-0.95) studies. For UC, physical activity was associated with a reduced risk in the observational cohort studies (RR 0.62; 95% CI, 0.43-0.88), but not in case-control studies (RR 0.74; 95% CI, 0.51-1.07).^64^

### 3.8 Body mass index

Obesity is linked to chronic low-grade inflammation, as excess adipose tissue produces pro-inflammatory cytokines such as tumor necrosis factor (TNF)-α and interleukin (IL)-6, contributing to immune dysregulation.^65^ A meta-analysis found an association of elevated body mass index (BMI) and CD (HR: 1.42; 95% CI, 1.18-1.71) but not UC (HR: 0.96; 95% CI, 0.80-1.14).^66^ Low BMI may also serve as an age-dependent risk factor for CD. Among individuals aged 18–40 years with normal weight in the Danish National Birth Cohort, low BMI was a stronger predictor of developing CD (adjusted HR: 1.8; 95% CI, 0.9-3.7) compared to obesity (HR: 1.5; 95% CI, 0.8-2.7).^67^ Interestingly, a meta-analysis of 4 studies revealed that patients treated with bariatric surgery for obesity had a slightly higher risk of subsequently developing IBD (OR: 1.17; 95% CI, 1.06-1.29) compared to a control group of obese patients who did not undergo surgery.^68^

### 3.9 Sleep

Poor sleep patterns are associated with elevated levels of pro-inflammatory cytokines, potentially mediated by gut dysbiosis, as demonstrated in animal model studies.^69^ A prospective study conducted by Ananthakrishnan et al. involving women enrolled in the NHS found that healthy sleep patterns may reduce the risk of developing IBD.^70^ Specifically, getting less than 6 hours (HR: 1.51; 95% CI, 1.10-2.09) or more than 9 hours (2.05, 95% CI, 1.44-1.92) of sleep per day was associated with an increased risk of developing UC. However, no significant correlation was observed between less than 6 hours (HR: 0.90, 95% CI, 0.63-1.28) or more than 9 hours (HR: 1.16, 95% CI, 0.73-1.83) of sleep and the risk of developing CD.^70^

### 3.10 Mental health

The increasing deterioration of mental health in modern life—driven by heightened stress, a fast-paced environment, mounting work and social pressures, and constant digital connectivity—underscores the significance of the mind-gut axis.^71^ Psychological distress activates the hypothalamic-pituitary-adrenal axis, leading to the release of corticotropin-releasing factor and adrenocorticotropic hormone, which have been shown to increase intestinal permeability and promote cytokine release.^71^ A systematic review demonstrated increased rates of depression in IBD.^72^ Frolkis et al. demonstrated that individuals with depression were more likely to develop UC (HR: 2.23; 95% CI, 1.92-2.60) and CD (HR: 2.11; 95% CI, 1.65-2.70).^73^ However, treating depression with anti-depressants mitigated the associated risk. A nested case-control study, which accounted for gastrointestinal symptoms preceding the diagnosis of IBD, found that depression was not significantly associated with the development of UC (OR: 1.13; 95% CI, 0.99-1.29) or CD (OR: 1.12; 95% CI, 0.91-1.38).^74^ Among other mental health disorders, schizophrenia may be associated with subsequent development of IBD (HR: 3.28; 95% CI, 2.49-4.33).^75^

### 3.11 Medications

Non steroidal anti-inflammatory drugs (NSAIDs) compromise the intestinal mucosal barrier, increase gut permeability, and activate inflammatory pathways through cyclooxygenase enzymes, leading to damage of the intestinal epithelium.^76^ Heavy use of NSAIDs has been positively associated with a later diagnosis of UC (HR: 1.87; 95% CI, 1.16-2.99) and CD (HR: 1.59; 95% CI, 0.99-2.56).^77^ While not all studies found an association between acetylsalicylic acid (ie, Aspirin) and IBD,^77^ a European cohort study of adults aged 30–74 found a correlation between regular aspirin use and increased risk of CD (OR: 6.14; 95% CI, 1.76-21.35).^78^

Reduced estrogen receptor beta mRNA expression precedes colitis in mouse models, suggesting that estrogen dysregulation may contribute to IBD development.^79^ A meta-analysis of 14 studies investigating the relationship between oral contraceptive use among women and IBD found an increased risk for developing CD (RR: 1.46; 95% CI, 1.26-1.70) and UC (RR: 1.28; 95% CI, 1.06-1.54) when controlling for smoking.^80^ A 2019 meta-analysis demonstrated an increased risk of developing UC with current oral contraceptive use (OR: 1.49; 95% CI, 1.12-1.96).^81^ A prospective cohort study showed hormone replacement therapy to be a risk factor for developing UC (current users, HR: 1.71; 95% CI, 1.07-2.74; past users, HR: 1.65; 95% CI, 1.03-2.66) but not CD (HR: 1.19; 95% CI, 0.78-1.82).^82^

Proton pump inhibitors (PPIs) may increase the risk of IBD by impairing intestinal permeability through disruption of tight junctions, increasing susceptibility to GI infections like C. difficile and altering gut microbiota composition.^83^ A meta-analysis of observational studies showed an increased risk of IBD in individuals previously exposed to or currently taking a proton pump inhibitor (aOR: 3.60; 95% CI, 1.10-11.74), primarily driven by CD, as the risk of UC was not significantly increased (aOR: 1.50; 95% CI, 0.5-4.5).^84^ In a pediatric cohort study, an increased risk of developing IBD was observed after at least one PPI prescription (OR: 3.6; 95% CI, 1.1-11.7), while no increased risk was found with H2 blocker medications (OR 1.6; 95% CI, 0.7-3.7).^85^

Antibiotic use has been associated with decreased diversity in the gut microbiome and incomplete recovery of the microbiome after discontinuation.^86^ A meta-analysis demonstrated that antibiotic exposure increased the risk of CD (OR: 1.74; 95% CI, 1.35-2.23) but not of UC (OR: 1.08; 95% CI, 0.91-1.27).^87^ Among pediatric populations, the association with CD was even stronger (OR: 2.75; 95% CI, 1.72-4.38).^87^ Antibiotic exposure during childhood was associated with a lifetime risk of CD (OR: 1.52; 95% CI, 1.23-1.87) but not UC (OR: 1.11; 95% CI, 0.93-1.33).^88^ A population-based case-control study found that antibiotic use within the first year of life increased the risk of developing both UC and CD.^89^ Another population-based case-control study demonstrated a dose-dependent relationship between antibiotic use and IBD onset, with CD being more common in cases with one or more disbursements, while UC was more common in cases with 3 or more disbursements.^90^

### 3.12 Appendectomy and tonsillectomy

The appendix and tonsils, part of the mucosa-associated lymphoid system, contribute to immune modulation and microbial defense, and their removal may impair immune tolerance, potentially increasing the risk of IBD.^91^ A recent meta-analysis found a higher risk of CD following tonsillectomy (OR: 1.93; 95% CI, 0.96-3.89) and appendectomy (OR: 1.57; 95% CI, 1.01-2.43). The risk of UC was increased after tonsillectomy (OR: 1.24; 95% CI, 1.18-1.30) but not appendectomy (OR: 0.60; 95% CI, 0.24-1.47).^92^

Previous meta-analyses have shown variable results. One meta-analysis showed lower odds for UC following appendectomy (OR: 0.44; 95% CI, 0.30-0.64),^93^ while another demonstrated an association between appendectomy and development of CD, with the risk being highest in the first year after surgery (RR: 6.69; 95% CI, 5.42-8.25) and returning to baseline after 5 years (RR: 1.08; 95% CI, 0.99-1.18). This association may be influenced by diagnostic bias, as the initial presentation of CD can sometimes mimic appendicitis.^94^

### 3.13 Latitude and vitamin D

Vitamin D regulates immunity by inhibiting Th1/Th17 cells, promoting IL-10-producing regulatory T cells, and influencing epithelial cells, antimicrobial peptides, and natural killer cells.^95^ A meta-analysis found that vitamin D deficiency was associated with an increased risk of IBD (OR: 1.64; 95% CI, 1.30-2.08).^96^ Vitamin D is obtained in part through sunlight, with its synthesis influenced by latitudinal variations in sunlight exposure. The NHS found that women residing in southern latitudes at age 30 had reduced odds of developing CD (OR: 0.48; 95% CI, 0.30-0.77) and UC (OR: 0.62; 95% CI, 0.42-0.90) in comparison with women in northern latitudes.^97^ A systematic review of population-based studies examining the pediatric incidence of CD, latitude, and ambient ultraviolet radiation found an increase in annual pediatric CD incidence of 0.23 new cases per 100 000 population in CD incidence in higher latitudes, particularly in regions with more months of low ultraviolet radiation.^98^

### 3.14 Urban vs rural living

Soon et al. conducted a meta-analysis on the association between urban living and IBD onset, demonstrating a positive relationship for both CD (incident rate ratio (IRR): 1.42; 95% CI, 1.26-1.60) and UC (IRR: 1.17; 95% CI, 1.03-1.32).^99^ A Canadian birth cohort study demonstrated a lower risk of IBD for people living in rural compared to urban households (IRR: 0.90; 95% CI, 0.81-0.99). Additionally, that study demonstrated that the protective relationships of rural living with IBD are strongest in the first 10 years of life (IRR: 0.58; 95% CI, 0.43-0.73).^100^ In early life, a meta-analysis demonstrated that urban living was associated with CD (OR: 1.45; 95% CI, 1.14-1.85) but not UC (OR: 1.16; 95% CI, 0.83-1.61).^101^

### 3.15 Pollution and greenspace

Air pollution has been associated with an increased risk of developing IBD. In mouse models, ingestion of particulate matter such as dust and smoke alters the gut microbiome and triggers inflammatory responses in the intestine.^102^ Residential exposure to nitrogen dioxide among individuals aged 23 years or younger has been associated with a higher risk of CD (OR: 2.31; 95% CI, 1.25-4.28), while exposure to sulfur dioxide among those aged 25 years or younger has been correlated with a higher risk of UC (OR: 2.00; 95% CI, 1.08-3.72).^103^ However, a case-control study based on European populations did not find significant relationships between IBD and various air pollutants.^104^ In pediatric-onset IBD, a study by Elten et al. demonstrated that the oxidant capacity of air pollutants was associated with an increased risk of developing IBD (HR: 1.08; 95% CI, 1.01-1.16).^105^

Greenspace, which refers to areas of vegetation or bodies of water in urban spaces that buffer neighborhoods from traffic, industry, and commercialization, has been associated with a reduced risk of IBD. A population-based study from Ontario, Canada, found that residential proximity to greenspace was linked to a lower hazard of pediatric-onset IBD (HR: 0.77; 95% CI, 0.74-0.81). The protective influence of greenspace on IBD risk may be due to reduced exposure to air pollution or healthier lifestyle factors, such as increased physical activity.^106^ Additionally, studies have implicated the ingestion of pollutants, including microplastics and organic pollutants like perfluoroalkyl substances, in the development of IBD.^107–109^

### 3.16 Challenges to studying environmental risk factors

Identifying consistent environmental risk factors for IBD has proven challenging, with many contradictory studies present in the literature.^110^ This difficulty partly stems from the approach taken by the scientific community in studying IBD.^111^ Several limitations in previous studies include the failure to account for the heterogeneity underlying IBD, imprecise quantification of the degree and timing of exposures, and the intrinsic biases inherent in observational research.^111^

Earlier research often relied on case-control studies, which are prone to biases such as recall errors, leading to exposure misclassification. While preclinical cohorts that collect environmental data before the diagnosis of IBD have minimized exposure misclassification, most of these prospective cohorts have focused on healthy adults. Consequently, these studies have often involved newly diagnosed IBD cases in older individuals, who may not represent the diverse spectrum of age at onset.^111^

Furthermore, much of the research on environmental risk factors has focused on populations of European ancestry. As IBD research expands to include more diverse populations, unique relationships between environmental determinants and IBD are being recognized. These collective limitations have contributed to the heterogeneity observed in the environmental health literature related to IBD.

## 4. Roadmap to translating environmental factor modification in preventing IBD

### 4.1 Predicting individuals at risk of IBD

Understanding the etiology of IBD involves deciphering the complex interplay between genetics, environmental factors, and the intestinal microbiome at various stages of life. With emerging epidemiological evidence, novel biomarkers of disease risk, and advanced prediction modeling techniques, we are increasingly able to identify behaviors and interventions that can modify the risk of IBD. While retrospective data can highlight potential exposure risks, identifying biological markers that predict future disease requires the study of prospective cohorts, particularly those focusing on individuals at risk before disease onset.

Several preclinical cohorts have identified biologic markers associated with the future development of IBD. The Crohn’s and Colitis Canada Genetic, Environmental, Microbial (CCC-GEM) project is a global prospective study that has recruited over 5000 healthy first-degree relatives of people with CD. This study assesses genetics, intestinal barrier function, immune function, and gut microbiome composition.^112^ The CCC-GEM project has identified markers such as higher antimicrobial serum antibody response^113^ and abnormal gut permeability^114^ as predictors of future CD, while increased fecal proteolytic activity has been associated with future onset of UC.^21^ Microbial communities present up to 5 years before the diagnosis of CD have also been linked to an increased risk of developing the disease.^115^

Preclinical cohorts, such as military serum banks, have identified serological markers such as anti-*Saccharomyces cerevisiae* mannan antibodies and perinuclear antineutrophil cytoplasm antibodies that predated the diagnosis of CD and UC, respectively.^116^ Autoantibodies to granulocyte macrophage-colony stimulating factor were detected in serum samples years prior to a CD diagnosis, particularly in individuals who later develop a more complex disease course.^117^ Additionally, proteomic biomarkers that predict both CD and UC years before disease onset have also been identified.^118–120^

The goal of identifying these risk factors is to distinguish between individuals at low risk and those at high risk, who may benefit from specific interventions, such as improving microbiome composition. For individuals at even higher risk, pharmaceutical interventions may be considered in future research. This approach is exemplified by randomized controlled trials of teplizumab, which slowed the onset of type 1 diabetes in high-risk relatives,^121^ and abatacept, which reduced the progression to rheumatoid arthritis in individuals with serum antibodies to citrullinated protein antigens and rheumatoid factor.^122^

### 4.2 Environmental risk factor modification in preventing IBD

The burden of IBD extends beyond individuals’ impact on healthcare systems and global economies. The economic implications are vast, encompassing hospitalization costs, lost workplace productivity, and premature retirement, among many other factors.^123^ Additionally, the ongoing cost of medications remains a significant concern for both individuals and healthcare providers.^124^ Therefore, strategies aimed at reducing the incidence of IBD have the potential to alleviate this burden, allowing for the intensification of resources for those living with the disease.^124^

Environmental modification strategies are crucial in preventing IBD and reducing its overall societal impact. Observational research has shown the potential for lifestyle and dietary changes to prevent the onset of IBD.^125^ For example, the incidence of CD could be reduced by 61.1% and UC by 42.2% among individuals who adhered to at least 7 of the following healthy living index domains: (1) minimal alcohol consumption (≤1 drink/day for women, and ≤2 drinks/day in men); (2) maintaining a BMI between 18.5 and 25 kg/m^2^; (3) engaging in ≥7.5 metabolic equivalent of task hours per week of physical activity; (4) never smoking; (5) eating ≥8 servings of fruit or vegetables per day; (6) ingesting ≥25 g of fiber per day; (7) consuming ≥0.5 servings of nuts or seeds per day; (8) Limiting red meat intake to <0.5 servings per day; and (9) eating ≥2 servings of fish per week. These findings have been validated in European cohorts, showing prevention rates ranging from 48.8% to 60.4% for CD and 46.8% to 56.3% for UC.^125^

Despite these promising observational data, interventional or randomized controlled studies assessing the efficacy of altering environmental risk factors are currently lacking. Furthermore, the impact of environmental risk factors may vary based on race, ethnicity, or geography, as evidenced by regional differences in the effect of smoking on CD.^60,111^ Therefore, future environmental studies must place greater emphasis on global representation to ensure that the outcomes of modifying environmental risk factors are generalizable across the diverse IBD population.

Nonetheless, the available evidence supports environmental health strategies that may reduce the likelihood of developing IBD. Box 1 provides guidance for healthcare providers when discussing potential actions to reduce the risk of IBD with their patients.^126^

Box 1: **Guidance for healthcare providers on reducing the risk of developing IBD based on meta-analyses of observational research studies, while awaiting higher quality interventional and randomized controlled studies of disease prevention.**

- Breastfeeding offers numerous health benefits to infants and is encouraged when possible. Breastfeeding for at least 3 months may be more beneficial than for shorter durations.^41^
- Certain dietary habits may be associated with a lower risk of IBD:
  - ◦ Lower intake of red meat and higher consumption of fish.^44,125^
  - ◦ Increase consumption of plant-based foods, including fruits and vegatables.^44,125^
  - ◦ Maintain the recommended daily fiber intake (>25 g/d).^125^
  - ◦ Ensure adequate vitamin D intake through diet or supplementation.^96^
  - ◦ Reduce consumption of ultra-processed food.^55,56^
  - ◦ Prioritize tea consumption while minimizing the intake of soft drinks.^51,52^
  - ◦ Adopt the Mediterranean-like diet^50^ over the traditional Western diet.
- Optimizing the following lifestyle factors may help prevent IBD:
  - ◦ Physical activity^63,125^: Follow the World Health Organization’s recommendations of at least 150 minutes of moderate-intensity aerobic physical activity or 75 minutes of vigorous-intensity aerobic physical activity per week for adults aged 18–64.
  - ◦ Maintaining a healthy weight^125,127^: Avoid obesity (defined as a BMI over 30) through regular exercise and healthy eating.
  - ◦ Mental health^72^: Individuals experiencing depression should seek appropriate treatment.
  - ◦ Smoking^58,80^: Avoid smoking. In addition to its negative impacts on cardiovascular health and its role as a risk factor for lung disease and numerous other concerns, smoking is also an independent risk factor for developing IBD. Smoking increases the risk of CD, while former smokers are at a higher risk of UC.
- Medications
  - ◦ PPIs: Avoid excessive use when possible.^77^
  - ◦ Antibiotics: Use antibiotics as recommended by healthcare providers. However, unnecessary use, particularly in early life, should be avoided.^87^
  - ◦ Oral contraceptive pills: Consult with a primary care physician on the risks and benefits of oral contraceptive pills compared to alternate forms of contraception.^128^

**Figure 3.**
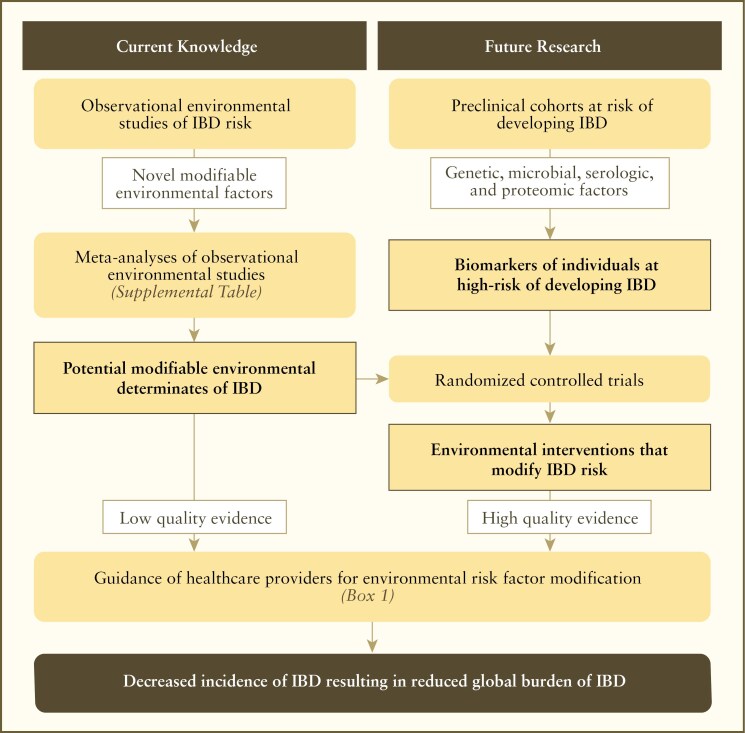
A framework for reducing the incidence of IBD that integrates current knowledge to support future interventional environmental health research, while providing guidance today to promote disease prevention in the future. IBD, inflammatory bowel disease.

Meta-analyses of observational cohort studies (see Supplemental Table) offer a list of candidate modifiable environmental risk factors. These factors provide current guidance to healthcare providers for potential risk modulation, albeit based on low-quality evidence (Box 1). Concurrently, research into preclinical cohorts is identifying biomarkers (genetic, serologic, microbial, and proteomic) that may help identify individuals at high risk for IBD. These high-risk populations can be targeted for randomized interventional clinical trials focused on disease prevention or delaying the onset of IBD, which will define high-quality evidence for future clinical guidance. The ultimate goal of this research is to develop a preventative health strategy that reduces the incidence and global burden of IBD (Figure 3).

## 5. Conclusion

The environmental determinates of IBD are multifactorial, with varying impacts across the age spectrum at the time of diagnosis. Many of these factors influence IBD through their effects on the intestinal microbiome, particularly during early life exposures. As preclinical cohorts continue to illuminate biomarkers—such as microbial, serologic, genetic, and proteomic indicators—high-risk individuals can be identified and targeted for interventional research. Modifying lifestyle and dietary factors presents a promising strategy for reducing the incidence of IBD. Ultimately, lowering the incidence of IBD is crucial in mitigating the rising global burden of IBD.

## Supplementary Material

jjaf042_suppl_Supplementary_Table_S1

## Data Availability

Data included were obtained from the results of publications that are cited within this manuscript.
