# Supplementary material for: Environmental risk factors of inflammatory bowel disease: toward a strategy of preventative health
Source: J Crohns Colitis. 2025 Mar 11;19(4):jjaf042. doi: 10.1093/ecco-jcc/jjaf042 (PMC12010164; doi:10.1093/ecco-jcc/jjaf042)
Supplement: jjaf042_suppl_Supplementary_Table_S1 [file jjaf042_suppl_supplementary_table_s1.docx]

Supplemental Material 1: Search Strategy

**Diet & inflammatory bowel disease**

Search: **diet AND inflammatory bowel disease risk** Filters: **Meta-Analysis**

(("diet"[MeSH Terms] OR "diet"[All Fields]) AND (("inflammatory bowel diseases"[MeSH Terms] OR ("inflammatory"[All Fields] AND "bowel"[All Fields] AND "diseases"[All Fields]) OR "inflammatory bowel diseases"[All Fields] OR ("inflammatory"[All Fields] AND "bowel"[All Fields] AND "disease"[All Fields]) OR "inflammatory bowel disease"[All Fields]) AND ("risk"[MeSH Terms] OR "risk"[All Fields]))) AND (meta-analysis[Filter])

**Findings: 35 publications**

**Processed food & inflammatory bowel disease**

Search: **processed food AND inflammatory bowel disease risk** Filters: **Meta-Analysis**

(("food, processed"[MeSH Terms] OR ("food"[All Fields] AND "processed"[All Fields]) OR "processed food"[All Fields] OR ("processed"[All Fields] AND "food"[All Fields])) AND (("inflammatory bowel diseases"[MeSH Terms] OR ("inflammatory"[All Fields] AND "bowel"[All Fields] AND "diseases"[All Fields]) OR "inflammatory bowel diseases"[All Fields] OR ("inflammatory"[All Fields] AND "bowel"[All Fields] AND "disease"[All Fields]) OR "inflammatory bowel disease"[All Fields]) AND ("risk"[MeSH Terms] OR "risk"[All Fields]))) AND (meta-analysis[Filter])

**Findings: 4 studies**

**Protein diet & inflammatory bowel disease**

Search: **"protein" AND inflammatory bowel disease risk** Filters: **Meta-Analysis**

("protein"[All Fields] AND (("inflammatory bowel diseases"[MeSH Terms] OR ("inflammatory"[All Fields] AND "bowel"[All Fields] AND "diseases"[All Fields]) OR "inflammatory bowel diseases"[All Fields] OR ("inflammatory"[All Fields] AND "bowel"[All Fields] AND "disease"[All Fields]) OR "inflammatory bowel disease"[All Fields]) AND ("risk"[MeSH Terms] OR "risk"[All Fields]))) AND (meta-analysis[Filter])

**Findings: 9 studies**

**Fiber & inflammatory bowel disease**

Search: **Fiber AND inflammatory bowel disease** Filters: **Meta-Analysis**

(("fiber"[All Fields] OR "fiber s"[All Fields] OR "fiberized"[All Fields] OR "fibers"[All Fields] OR "fibre"[All Fields] OR "fibre s"[All Fields] OR "fibres"[All Fields]) AND ("inflammatory bowel diseases"[MeSH Terms] OR ("inflammatory"[All Fields] AND "bowel"[All Fields] AND "diseases"[All Fields]) OR "inflammatory bowel diseases"[All Fields] OR ("inflammatory"[All Fields] AND "bowel"[All Fields] AND "disease"[All Fields]) OR "inflammatory bowel disease"[All Fields])) AND (meta-analysis[Filter])

**Findings: 12 studies**

**Western diet & inflammatory bowel disease**

Search: **Western Diet AND inflammatory bowel disease** Filters: **Meta-Analysis**

(("diet, western"[MeSH Terms] OR ("diet"[All Fields] AND "western"[All Fields]) OR "western diet"[All Fields] OR ("western"[All Fields] AND "diet"[All Fields])) AND ("inflammatory bowel diseases"[MeSH Terms] OR ("inflammatory"[All Fields] AND "bowel"[All Fields] AND "diseases"[All Fields]) OR "inflammatory bowel diseases"[All Fields] OR ("inflammatory"[All Fields] AND "bowel"[All Fields] AND "disease"[All Fields]) OR "inflammatory bowel disease"[All Fields])) AND (meta-analysis[Filter])

**Findings: 2 studies**

**Polyunsaturated fatty acids & inflammatory bowel disease**

Search: **polyunsaturated fatty acids AND inflammatory bowel disease** Filters: **Meta-Analysis**

(("fatty acids, unsaturated"[MeSH Terms] OR ("fatty"[All Fields] AND "acids"[All Fields] AND "unsaturated"[All Fields]) OR "unsaturated fatty acids"[All Fields] OR ("polyunsaturated"[All Fields] AND "fatty"[All Fields] AND "acids"[All Fields]) OR "polyunsaturated fatty acids"[All Fields]) AND ("inflammatory bowel diseases"[MeSH Terms] OR ("inflammatory"[All Fields] AND "bowel"[All Fields] AND "diseases"[All Fields]) OR "inflammatory bowel diseases"[All Fields] OR ("inflammatory"[All Fields] AND "bowel"[All Fields] AND "disease"[All Fields]) OR "inflammatory bowel disease"[All Fields])) AND (meta-analysis[Filter])

**Findings: 13 studies**

**Carbohydrate intake & inflammatory bowel disease**

Search: **carbohydrate intake AND inflammatory bowel disease** Filters: **Meta-Analysis**

(("carbohydrate s"[All Fields] OR "carbohydrated"[All Fields] OR "carbohydrates"[MeSH Terms] OR "carbohydrates"[All Fields] OR "carbohydrate"[All Fields]) AND ("intake"[All Fields] OR "intake s"[All Fields] OR "intakes"[All Fields]) AND ("inflammatory bowel diseases"[MeSH Terms] OR ("inflammatory"[All Fields] AND "bowel"[All Fields] AND "diseases"[All Fields]) OR "inflammatory bowel diseases"[All Fields] OR ("inflammatory"[All Fields] AND "bowel"[All Fields] AND "disease"[All Fields]) OR "inflammatory bowel disease"[All Fields])) AND (meta-analysis[Filter])

**Findings: 6 studies**

**Beverage & inflammatory bowel disease**

Search: **beverage AND inflammatory bowel disease** Filters: **Meta-Analysis**

(("beverage s"[All Fields] OR "beverages"[MeSH Terms] OR "beverages"[All Fields] OR "beverage"[All Fields]) AND ("inflammatory bowel diseases"[MeSH Terms] OR ("inflammatory"[All Fields] AND "bowel"[All Fields] AND "diseases"[All Fields]) OR "inflammatory bowel diseases"[All Fields] OR ("inflammatory"[All Fields] AND "bowel"[All Fields] AND "disease"[All Fields]) OR "inflammatory bowel disease"[All Fields])) AND (meta-analysis[Filter])

**Findings: 5 studies**

**Macronutrient & inflammatory bowel disease**

Search: **macronutrient AND inflammatory bowel disease** Filters: **Meta-Analysis**

(("nutrients"[MeSH Terms] OR "nutrients"[All Fields] OR "macronutrient"[All Fields] OR "macronutrients"[All Fields]) AND ("inflammatory bowel diseases"[MeSH Terms] OR ("inflammatory"[All Fields] AND "bowel"[All Fields] AND "diseases"[All Fields]) OR "inflammatory bowel diseases"[All Fields] OR ("inflammatory"[All Fields] AND "bowel"[All Fields] AND "disease"[All Fields]) OR "inflammatory bowel disease"[All Fields])) AND (meta-analysis[Filter])

**Findings: 31 studies**

**Fat & inflammatory bowel disease**

Search: **fat AND inflammatory bowel disease** Filters: **Meta-Analysis**

("fat"[All Fields] AND ("inflammatory bowel diseases"[MeSH Terms] OR ("inflammatory"[All Fields] AND "bowel"[All Fields] AND "diseases"[All Fields]) OR "inflammatory bowel diseases"[All Fields] OR ("inflammatory"[All Fields] AND "bowel"[All Fields] AND "disease"[All Fields]) OR "inflammatory bowel disease"[All Fields])) AND (meta-analysis[Filter])

**Findings: 15 studies**

**Meat & inflammatory bowel disease**

Search: **meat AND inflammatory bowel disease** Filters: **Meta-Analysis**

(("meat"[MeSH Terms] OR "meat"[All Fields]) AND ("inflammatory bowel diseases"[MeSH Terms] OR ("inflammatory"[All Fields] AND "bowel"[All Fields] AND "diseases"[All Fields]) OR "inflammatory bowel diseases"[All Fields] OR ("inflammatory"[All Fields] AND "bowel"[All Fields] AND "disease"[All Fields]) OR "inflammatory bowel disease"[All Fields])) AND (meta-analysis[Filter])

**Findings: 11 studies**

**Microbiota & inflammatory bowel disease**

Search: **microbiome AND inflammatory bowel disease** Filters: **Meta-Analysis**

(("microbiome s"[All Fields] OR "microbiomic"[All Fields] OR "microbiomics"[All Fields] OR "microbiota"[MeSH Terms] OR "microbiota"[All Fields] OR "microbiome"[All Fields] OR "microbiomes"[All Fields]) AND ("inflammatory bowel diseases"[MeSH Terms] OR ("inflammatory"[All Fields] AND "bowel"[All Fields] AND "diseases"[All Fields]) OR "inflammatory bowel diseases"[All Fields] OR ("inflammatory"[All Fields] AND "bowel"[All Fields] AND "disease"[All Fields]) OR "inflammatory bowel disease"[All Fields])) AND (meta-analysis[Filter])

**Findings: 100 studies**

**Helicobacter & inflammatory bowel disease**

Search: **helicobacter AND inflammatory bowel disease** Filters: **Meta-Analysis**

(("helicobacter"[MeSH Terms] OR "helicobacter"[All Fields] OR "helicobacters"[All Fields]) AND ("inflammatory bowel diseases"[MeSH Terms] OR ("inflammatory"[All Fields] AND "bowel"[All Fields] AND "diseases"[All Fields]) OR "inflammatory bowel diseases"[All Fields] OR ("inflammatory"[All Fields] AND "bowel"[All Fields] AND "disease"[All Fields]) OR "inflammatory bowel disease"[All Fields])) AND (meta-analysis[Filter])

**Findings: 12 studies**

**Escherichia coli & inflammatory bowel disease**

Search: **Escherichia coli AND inflammatory bowel disease** Filters: **Meta-Analysis**

(("escherichia coli"[MeSH Terms] OR ("escherichia"[All Fields] AND "coli"[All Fields]) OR "escherichia coli"[All Fields]) AND ("inflammatory bowel diseases"[MeSH Terms] OR ("inflammatory"[All Fields] AND "bowel"[All Fields] AND "diseases"[All Fields]) OR "inflammatory bowel diseases"[All Fields] OR ("inflammatory"[All Fields] AND "bowel"[All Fields] AND "disease"[All Fields]) OR "inflammatory bowel disease"[All Fields])) AND (meta-analysis[Filter])

**Findings: 9 studies**

**Faecalibacterium & inflammatory bowel disease**

Search: **Faecalibacterium AND inflammatory bowel disease** Filters: **Meta-Analysis**

(("faecalibacterium"[MeSH Terms] OR "faecalibacterium"[All Fields]) AND ("inflammatory bowel diseases"[MeSH Terms] OR ("inflammatory"[All Fields] AND "bowel"[All Fields] AND "diseases"[All Fields]) OR "inflammatory bowel diseases"[All Fields] OR ("inflammatory"[All Fields] AND "bowel"[All Fields] AND "disease"[All Fields]) OR "inflammatory bowel disease"[All Fields])) AND (meta-analysis[Filter])

**Findings: 3 studies**

**Campylobacter & inflammatory bowel disease**

Search: **Campylobacter AND inflammatory bowel disease** Filters: **Meta-Analysis**

**Findings: 1 study**

**Cytomegalovirus & inflammatory bowel disease**

Search: **Cytomegalovirus AND inflammatory bowel disease** Filters: **Meta-Analysis**

(("cytomegalovirus"[MeSH Terms] OR "cytomegalovirus"[All Fields] OR "cytomegaloviruses"[All Fields]) AND ("inflammatory bowel diseases"[MeSH Terms] OR ("inflammatory"[All Fields] AND "bowel"[All Fields] AND "diseases"[All Fields]) OR "inflammatory bowel diseases"[All Fields] OR ("inflammatory"[All Fields] AND "bowel"[All Fields] AND "disease"[All Fields]) OR "inflammatory bowel disease"[All Fields])) AND (meta-analysis[Filter])

**Findings: 10 studies**

**Mycobacterium & inflammatory bowel disease**

Search: **Mycobacterium AND inflammatory bowel disease** Filters: **Meta-Analysis**

(("mycobacteriae"[All Fields] OR "mycobacterias"[All Fields] OR "mycobacterium"[MeSH Terms] OR "mycobacterium"[All Fields] OR "mycobacteria"[All Fields]) AND ("inflammatory bowel diseases"[MeSH Terms] OR ("inflammatory"[All Fields] AND "bowel"[All Fields] AND "diseases"[All Fields]) OR "inflammatory bowel diseases"[All Fields] OR ("inflammatory"[All Fields] AND "bowel"[All Fields] AND "disease"[All Fields]) OR "inflammatory bowel disease"[All Fields])) AND (meta-analysis[Filter])

**Findings: 9 studies**

**Virus & inflammatory bowel disease**

Search: **virus AND inflammatory bowel disease** Filters: **Meta-Analysis**

(("virology"[MeSH Subheading] OR "virology"[All Fields] OR "viruses"[All Fields] OR "viruses"[MeSH Terms] OR "virus s"[All Fields] OR "viruse"[All Fields] OR "virus"[All Fields]) AND ("inflammatory bowel diseases"[MeSH Terms] OR ("inflammatory"[All Fields] AND "bowel"[All Fields] AND "diseases"[All Fields]) OR "inflammatory bowel diseases"[All Fields] OR ("inflammatory"[All Fields] AND "bowel"[All Fields] AND "disease"[All Fields]) OR "inflammatory bowel disease"[All Fields])) AND (meta-analysis[Filter])

**Findings: 38 studies**

**Antibiotics & inflammatory bowel disease**

Search: **antibiotics AND inflammatory bowel disease** Filters: **Meta-Analysis**

(("anti bacterial agents"[Pharmacological Action] OR "anti bacterial agents"[MeSH Terms] OR ("anti bacterial"[All Fields] AND "agents"[All Fields]) OR "anti bacterial agents"[All Fields] OR "antibiotic"[All Fields] OR "antibiotics"[All Fields] OR "antibiotic s"[All Fields] OR "antibiotical"[All Fields]) AND ("inflammatory bowel diseases"[MeSH Terms] OR ("inflammatory"[All Fields] AND "bowel"[All Fields] AND "diseases"[All Fields]) OR "inflammatory bowel diseases"[All Fields] OR ("inflammatory"[All Fields] AND "bowel"[All Fields] AND "disease"[All Fields]) OR "inflammatory bowel disease"[All Fields])) AND (meta-analysis[Filter])

**Findings: 68 studies**

**Isotretinoin & inflammatory bowel disease**

Search: **isotretinoin AND inflammatory bowel disease** Filters: **Meta-Analysis**

(("isotretinoin"[MeSH Terms] OR "isotretinoin"[All Fields]) AND ("inflammatory bowel diseases"[MeSH Terms] OR ("inflammatory"[All Fields] AND "bowel"[All Fields] AND "diseases"[All Fields]) OR "inflammatory bowel diseases"[All Fields] OR ("inflammatory"[All Fields] AND "bowel"[All Fields] AND "disease"[All Fields]) OR "inflammatory bowel disease"[All Fields])) AND (meta-analysis[Filter])

**Findings: 4 studies**

**Proton pump inhibitors & inflammatory bowel disease**

Search: **proton pump inhibitors AND inflammatory bowel disease** Filters: **Meta-Analysis**

(("proton pump inhibitors"[Pharmacological Action] OR "proton pump inhibitors"[MeSH Terms] OR ("proton"[All Fields] AND "pump"[All Fields] AND "inhibitors"[All Fields]) OR "proton pump inhibitors"[All Fields]) AND ("inflammatory bowel diseases"[MeSH Terms] OR ("inflammatory"[All Fields] AND "bowel"[All Fields] AND "diseases"[All Fields]) OR "inflammatory bowel diseases"[All Fields] OR ("inflammatory"[All Fields] AND "bowel"[All Fields] AND "disease"[All Fields]) OR "inflammatory bowel disease"[All Fields])) AND (meta-analysis[Filter])

**Findings: 4 studies**

**Contraceptives & inflammatory bowel disease**

Search: **contraceptives AND inflammatory bowel disease** Filters: **Meta-Analysis**

(("contracept"[All Fields] OR "contracepted"[All Fields] OR "contracepting"[All Fields] OR "contraception"[MeSH Terms] OR "contraception"[All Fields] OR "contraceptions"[All Fields] OR "contraceptive agents"[Pharmacological Action] OR "contraceptive agents"[MeSH Terms] OR ("contraceptive"[All Fields] AND "agents"[All Fields]) OR "contraceptive agents"[All Fields] OR "contraceptives"[All Fields] OR "contraceptive devices"[MeSH Terms] OR ("contraceptive"[All Fields] AND "devices"[All Fields]) OR "contraceptive devices"[All Fields] OR "contraceptive"[All Fields] OR "contraceptive s"[All Fields] OR "contraceptively"[All Fields]) AND ("inflammatory bowel diseases"[MeSH Terms] OR ("inflammatory"[All Fields] AND "bowel"[All Fields] AND "diseases"[All Fields]) OR "inflammatory bowel diseases"[All Fields] OR ("inflammatory"[All Fields] AND "bowel"[All Fields] AND "disease"[All Fields]) OR "inflammatory bowel disease"[All Fields])) AND (meta-analysis[Filter])

**Findings: 10 studies**

**Cesarean delivery & inflammatory bowel disease**

Search: **Cesarean delivery AND inflammatory bowel disease** Filters: **Meta-Analysis**

(("cesarean section"[MeSH Terms] OR ("cesarean"[All Fields] AND "section"[All Fields]) OR "cesarean section"[All Fields] OR ("cesarean"[All Fields] AND "delivery"[All Fields]) OR "cesarean delivery"[All Fields]) AND ("inflammatory bowel diseases"[MeSH Terms] OR ("inflammatory"[All Fields] AND "bowel"[All Fields] AND "diseases"[All Fields]) OR "inflammatory bowel diseases"[All Fields] OR ("inflammatory"[All Fields] AND "bowel"[All Fields] AND "disease"[All Fields]) OR "inflammatory bowel disease"[All Fields])) AND (meta-analysis[Filter])

**Findings 4 studies**

**Periodontitis & inflammatory bowel disease**

Search: **periodontitis AND inflammatory bowel disease** Filters: **Meta-Analysis**

(("periodontal"[All Fields] OR "periodontally"[All Fields] OR "periodontically"[All Fields] OR "periodontics"[MeSH Terms] OR "periodontics"[All Fields] OR "periodontic"[All Fields] OR "periodontitis"[MeSH Terms] OR "periodontitis"[All Fields] OR "periodontitides"[All Fields]) AND ("inflammatory bowel diseases"[MeSH Terms] OR ("inflammatory"[All Fields] AND "bowel"[All Fields] AND "diseases"[All Fields]) OR "inflammatory bowel diseases"[All Fields] OR ("inflammatory"[All Fields] AND "bowel"[All Fields] AND "disease"[All Fields]) OR "inflammatory bowel disease"[All Fields])) AND (meta-analysis[Filter])

**Findings: 9 studies**

**Urban & inflammatory bowel disease**

Search: **urban AND inflammatory bowel disease** Filters: **Meta-Analysis**

(("urban"[All Fields] OR "urbanicity"[All Fields] OR "urbanism"[All Fields] OR "urbanity"[All Fields] OR "urbanization"[MeSH Terms] OR "urbanization"[All Fields] OR "urbanizations"[All Fields] OR "urbanize"[All Fields] OR "urbanized"[All Fields] OR "urbanizes"[All Fields] OR "urbanizing"[All Fields] OR "urbans"[All Fields]) AND ("inflammatory bowel diseases"[MeSH Terms] OR ("inflammatory"[All Fields] AND "bowel"[All Fields] AND "diseases"[All Fields]) OR "inflammatory bowel diseases"[All Fields] OR ("inflammatory"[All Fields] AND "bowel"[All Fields] AND "disease"[All Fields]) OR "inflammatory bowel disease"[All Fields])) AND (meta-analysis[Filter])

**Findings: 4 studies**

**Breastfeeding & inflammatory bowel disease**

Search: **breastfeeding AND inflammatory bowel disease** Filters: **Meta-Analysis**

(("breast feeding"[MeSH Terms] OR ("breast"[All Fields] AND "feeding"[All Fields]) OR "breast feeding"[All Fields] OR "breastfeeding"[All Fields] OR "breastfeedings"[All Fields] OR "breastfeeders"[All Fields]) AND ("inflammatory bowel diseases"[MeSH Terms] OR ("inflammatory"[All Fields] AND "bowel"[All Fields] AND "diseases"[All Fields]) OR "inflammatory bowel diseases"[All Fields] OR ("inflammatory"[All Fields] AND "bowel"[All Fields] AND "disease"[All Fields]) OR "inflammatory bowel disease"[All Fields])) AND (meta-analysis[Filter])

**Findings: 6 studies**

**Obesity & inflammatory bowel disease**

Search: **obesity AND inflammatory bowel disease** Filters: **Meta-Analysis**

(("obeses"[All Fields] OR "obesity"[MeSH Terms] OR "obesity"[All Fields] OR "obese"[All Fields] OR "obesities"[All Fields] OR "obesity s"[All Fields]) AND ("inflammatory bowel diseases"[MeSH Terms] OR ("inflammatory"[All Fields] AND "bowel"[All Fields] AND "diseases"[All Fields]) OR "inflammatory bowel diseases"[All Fields] OR ("inflammatory"[All Fields] AND "bowel"[All Fields] AND "disease"[All Fields]) OR "inflammatory bowel disease"[All Fields])) AND (meta-analysis[Filter])

**Findings: 31 studies**

**Exercise & inflammatory bowel disease**

Search: **exercise AND inflammatory bowel disease** Filters: **Meta-Analysis**

(("exercise"[MeSH Terms] OR "exercise"[All Fields] OR "exercises"[All Fields] OR "exercise therapy"[MeSH Terms] OR ("exercise"[All Fields] AND "therapy"[All Fields]) OR "exercise therapy"[All Fields] OR "exercising"[All Fields] OR "exercise s"[All Fields] OR "exercised"[All Fields] OR "exerciser"[All Fields] OR "exercisers"[All Fields]) AND ("inflammatory bowel diseases"[MeSH Terms] OR ("inflammatory"[All Fields] AND "bowel"[All Fields] AND "diseases"[All Fields]) OR "inflammatory bowel diseases"[All Fields] OR ("inflammatory"[All Fields] AND "bowel"[All Fields] AND "disease"[All Fields]) OR "inflammatory bowel disease"[All Fields])) AND (meta-analysis[Filter])

**Findings: 14 studies**

**Appendicectomy & inflammatory bowel disease**

Search: **appendicectomy AND inflammatory bowel disease** Filters: **Meta-Analysis**

(("appendectomy"[MeSH Terms] OR "appendectomy"[All Fields] OR "appendectomies"[All Fields] OR "appendicectomies"[All Fields] OR "appendicectomy"[All Fields]) AND ("inflammatory bowel diseases"[MeSH Terms] OR ("inflammatory"[All Fields] AND "bowel"[All Fields] AND "diseases"[All Fields]) OR "inflammatory bowel diseases"[All Fields] OR ("inflammatory"[All Fields] AND "bowel"[All Fields] AND "disease"[All Fields]) OR "inflammatory bowel disease"[All Fields])) AND (meta-analysis[Filter])

**Findings: 19 studies**

**Bariatric surgery & inflammatory bowel disease**

Search: **bariatric surgery AND inflammatory bowel disease** Filters: **Meta-Analysis**

(("bariatric surgery"[MeSH Terms] OR ("bariatric"[All Fields] AND "surgery"[All Fields]) OR "bariatric surgery"[All Fields]) AND ("inflammatory bowel diseases"[MeSH Terms] OR ("inflammatory"[All Fields] AND "bowel"[All Fields] AND "diseases"[All Fields]) OR "inflammatory bowel diseases"[All Fields] OR ("inflammatory"[All Fields] AND "bowel"[All Fields] AND "disease"[All Fields]) OR "inflammatory bowel disease"[All Fields])) AND (meta-analysis[Filter])

**Findings: 5 studies**

**Smoking & inflammatory bowel disease**

Search: **smoking AND inflammatory bowel disease** Filters: **Meta-Analysis**

(("smoke"[MeSH Terms] OR "smoke"[All Fields] OR "smoke s"[All Fields] OR "smoked"[All Fields] OR "smokes"[All Fields] OR "smoking"[MeSH Terms] OR "smoking"[All Fields] OR "smokings"[All Fields] OR "smoking s"[All Fields]) AND ("inflammatory bowel diseases"[MeSH Terms] OR ("inflammatory"[All Fields] AND "bowel"[All Fields] AND "diseases"[All Fields]) OR "inflammatory bowel diseases"[All Fields] OR ("inflammatory"[All Fields] AND "bowel"[All Fields] AND "disease"[All Fields]) OR "inflammatory bowel disease"[All Fields])) AND (meta-analysis[Filter])

**Findings: 48 studies**

**Vitamin D & inflammatory bowel disease**

Search: **vitamin D AND inflammatory bowel disease** Filters: **Meta-Analysis**

(("vitamin d"[MeSH Terms] OR "vitamin d"[All Fields] OR "ergocalciferols"[MeSH Terms] OR "ergocalciferols"[All Fields]) AND ("inflammatory bowel diseases"[MeSH Terms] OR ("inflammatory"[All Fields] AND "bowel"[All Fields] AND "diseases"[All Fields]) OR "inflammatory bowel diseases"[All Fields] OR ("inflammatory"[All Fields] AND "bowel"[All Fields] AND "disease"[All Fields]) OR "inflammatory bowel disease"[All Fields])) AND (meta-analysis[Filter])

**Findings: 27 studies**

**Tonsillectomy & inflammatory bowel disease**

Search: **tonsillectomy AND inflammatory bowel disease** Filters: **Meta-Analysis**

(("tonsillectomy"[MeSH Terms] OR "tonsillectomy"[All Fields] OR "tonsillectomies"[All Fields]) AND ("inflammatory bowel diseases"[MeSH Terms] OR ("inflammatory"[All Fields] AND "bowel"[All Fields] AND "diseases"[All Fields]) OR "inflammatory bowel diseases"[All Fields] OR ("inflammatory"[All Fields] AND "bowel"[All Fields] AND "disease"[All Fields]) OR "inflammatory bowel disease"[All Fields])) AND (meta-analysis[Filter])

**Findings: 4 studies**

**Antidepressants & inflammatory bowel disease**

Search: **antidepressants AND inflammatory bowel disease** Filters: **Meta-Analysis**

(("inflammatory bowel diseases"[MeSH Terms] OR ("inflammatory"[All Fields] AND "bowel"[All Fields] AND "diseases"[All Fields]) OR "inflammatory bowel diseases"[All Fields] OR ("inflammatory"[All Fields] AND "bowel"[All Fields] AND "disease"[All Fields]) OR "inflammatory bowel disease"[All Fields]) AND ("antidepressent"[All Fields] OR "antidepression"[All Fields] OR "antidepressive agents"[Pharmacological Action] OR "antidepressive agents"[MeSH Terms] OR ("antidepressive"[All Fields] AND "agents"[All Fields]) OR "antidepressive agents"[All Fields] OR "antidepressant"[All Fields] OR "antidepressants"[All Fields] OR "antidepressive"[All Fields] OR "antidepressives"[All Fields])) AND (meta-analysis[Filter])

**Finding: 4 studies**

**Anxiolytics & inflammatory bowel disease**

Search: **anxiolytics AND inflammatory bowel disease** Filters: **Meta-Analysis**

(("inflammatory bowel diseases"[MeSH Terms] OR ("inflammatory"[All Fields] AND "bowel"[All Fields] AND "diseases"[All Fields]) OR "inflammatory bowel diseases"[All Fields] OR ("inflammatory"[All Fields] AND "bowel"[All Fields] AND "disease"[All Fields]) OR "inflammatory bowel disease"[All Fields]) AND ("anti anxiety agents"[Pharmacological Action] OR "anti anxiety agents"[MeSH Terms] OR ("anti anxiety"[All Fields] AND "agents"[All Fields]) OR "anti anxiety agents"[All Fields] OR "anxiolytic"[All Fields] OR "anxiolytics"[All Fields])) AND (meta-analysis[Filter])

**Finding: 2 studies**

**Visceral fat & inflammatory bowel disease**

Search: **visceral fat AND inflammatory bowel disease risk** Filters: **Meta-Analysis**

(("inflammatory bowel diseases"[MeSH Terms] OR ("inflammatory"[All Fields] AND "bowel"[All Fields] AND "diseases"[All Fields]) OR "inflammatory bowel diseases"[All Fields] OR ("inflammatory"[All Fields] AND "bowel"[All Fields] AND "disease"[All Fields]) OR "inflammatory bowel disease"[All Fields]) AND ("risk"[MeSH Terms] OR "risk"[All Fields]) AND ("intra abdominal fat"[MeSH Terms] OR ("intra abdominal"[All Fields] AND "fat"[All Fields]) OR "intra abdominal fat"[All Fields] OR ("visceral"[All Fields] AND "fat"[All Fields]) OR "visceral fat"[All Fields])) AND (meta-analysis[Filter])

**Findings: 0 studies**

**Alcohol & inflammatory bowel disease**

Search: **alcohol AND inflammatory bowel disease** Filters: **Meta-Analysis**

(("inflammatory bowel diseases"[MeSH Terms] OR ("inflammatory"[All Fields] AND "bowel"[All Fields] AND "diseases"[All Fields]) OR "inflammatory bowel diseases"[All Fields] OR ("inflammatory"[All Fields] AND "bowel"[All Fields] AND "disease"[All Fields]) OR "inflammatory bowel disease"[All Fields]) AND ("alcohol s"[All Fields] OR "alcoholate"[All Fields] OR "alcoholates"[All Fields] OR "alcohols"[MeSH Terms] OR "alcohols"[All Fields] OR "ethanol"[MeSH Terms] OR "ethanol"[All Fields] OR "alcohol"[All Fields])) AND (meta-analysis[Filter])

**Findings: 21 studies**

**Sleep & inflammatory bowel disease**

Search: **sleep AND inflammatory bowel disease** Filters: **Meta-Analysis**

(("sleep"[MeSH Terms] OR "sleep"[All Fields] OR "sleeping"[All Fields] OR "sleeps"[All Fields] OR "sleep s"[All Fields]) AND ("inflammatory bowel diseases"[MeSH Terms] OR ("inflammatory"[All Fields] AND "bowel"[All Fields] AND "diseases"[All Fields]) OR "inflammatory bowel diseases"[All Fields] OR ("inflammatory"[All Fields] AND "bowel"[All Fields] AND "disease"[All Fields]) OR "inflammatory bowel disease"[All Fields])) AND (meta-analysis[Filter])

**Findings: 10 studies**

**NSAIDS & Inflammatory bowel disease**

Search: **NSAIDs AND inflammatory bowel disease** Filters: **Meta-Analysis**

(("anti inflammatory agents non steroidal"[Pharmacological Action] OR "anti inflammatory agents, non steroidal"[MeSH Terms] OR ("anti inflammatory"[All Fields] AND "agents"[All Fields] AND "non steroidal"[All Fields]) OR "non-steroidal anti-inflammatory agents"[All Fields] OR "nsaid"[All Fields] OR "nsaids"[All Fields] OR "nsaid s"[All Fields]) AND ("inflammatory bowel diseases"[MeSH Terms] OR ("inflammatory"[All Fields] AND "bowel"[All Fields] AND "diseases"[All Fields]) OR "inflammatory bowel diseases"[All Fields] OR ("inflammatory"[All Fields] AND "bowel"[All Fields] AND "disease"[All Fields]) OR "inflammatory bowel disease"[All Fields])) AND (meta-analysis[Filter])

**Findings: 147 studies**

**TOTAL: 766 systematic reviews and meta-analysis**

**Duplicated: 210**

**Not meta-analysis or not of the interest of the topic: 477**

**FINAL INCLUSION: 79**

# Supplemental Table 1: Potentially Modifiable Environmental Risk Factors of IBD: Published Systematic Reviews and Meta-Analyses. The content of this table is updated monthly and can be freely accessed at [www.ibd-eii.com/ibdmultifactorial/](http://www.ibd-eii.com/ibdmultifactorial/).

| **Reference** | **Year** | **Factor analysed** | **Studies included** | **Pooled Random effects with 95% Confidence Intervals** |
| --- | --- | --- | --- | --- |
| **EARLY LIFE EXPOSURE** | | | | |
| Agrawal M et al.^1^ | 2021 | Early life exposures  *pOR (pooled OR) | 39  9  4  4  10  11  7  12  2  3 | IBD risk:   - Maternal smoking: **(pOR: 1.49; 1.17, 1.90; *I*^2^=72%)** - Maternal age: (OR: 0.86; 0.45, 1.65; *I*^2^=87.1%) - Antibiotic during pregnancy: **(OR: 1.75; 1.22, 2.51; *I*^2^=0%)** - Low birth weight: (pOR: 0.92; 0.72, 1.16; *I*^2^=76.8%) - Premature birth: (pOR: 1.06; 0.93, 1.19; *I*^2^=0%) - Rural vs urban living: (pOR: 1.03; 0.81, 1.31; *I*^2^=52.8%) - Vaccination: (OR: 1.08; 0.91, 1.28; *I*^2^=55.5%) - Otitis media between 0–5 years: **(OR: 2.11; 1.22, 3.62; *I*^2^=36.9%)**   CD risk:   - Maternal smoking: (pOR: 1.21; 0.75, 1.96; *I*^2^=83.55%) - Maternal age: (OR: 0.77; 0.22, 2.73; *I*^2^=95.27%) - Low birth weight: (pOR: 0.86; 0.62, 1.23; *I*^2^=70.7%) - Premature birth: (pOR: 1.07; 0.89, 1.29; *I*^2^=5.3%) - Rural vs urban living: (pOR: 1.15; 0.67, 1.98; *I*^2^=68.8%) - Vaccination: (OR: 1.06; 0.82, 1.37; *I*^2^=64.7%) - Otitis media between 0–5 years: **(OR: 1.95; 1.20, 3.17; *I*^2^=4.4%)** - Passive smoking: (OR: 1.08; 0.95, 1.2; *I*^2^=0%)   UC risk:   - Maternal smoking: (pOR: 1.51; 0.99, 2.31; *I*^2^=68.05%) - Low birth weight: (pOR: 0.83; 0.41, 1.68; *I*^2^=81.7%) - Premature birth: (pOR: 1.02; 0.67, 1.57; *I*^2^=53.6%) - Rural vs urban living: (pOR: 1.05; 0.84, 1.32; *I*^2^=38.3%) - Vaccination: (OR: 1.05; 0.84, 1.30; *I*^2^=28.2%) |
| Cholapranee A et al.^2^ | 2016 | Environmental Hygiene Factors   - Exposure to pet/farm animals - Bedroom & home sharing - Personal toilet and hot water   Number of siblings | 15  21  10  8 | IBD risk:   - Exposure to pet: **(OR: 0.76; 0.63, 0.88)*** - Farm animals: **(OR: 0.45; 0.29, 0.62)*** - Sharing home: **(OR: 0.53; 0.30, 0.76)*** - Bed sharing: **(OR: 0.66; 0.46, 0.87)*** - Personal toilet: **(OR: 0.82; 0.69, 0.95)*** - Hot water: (OR: 0.86; 0.71, 1.01)***** - Greater number of siblings: (OR: 0.95; 0.84, 1.06)*****   CD risk   - Exposure to pet: **(OR: 0.77; 0.59, 0.94)*** - Farm animals: **(OR: 0.46; 0.20, 0.72)*** - Sharing home: **(OR: 0.49; 0.25, 0.75)*** - Bed sharing: **(OR: 0.54; 0.43, 0.65)** ***** - Personal toilet: (OR: 1.11; 0.81, 1.40)***** - Hot water: (OR: 1.00; 0.73, 1.27)***** - Greater number of siblings: **(OR: 0.93; 0.88, 0.98)***   UC risk:   - Exposure to pet: **(OR: 0.75; 0.56, 0.94)*** - Farm animals: **(OR: 0.44; 0.14, 0.74)*** - Sharing home: (OR: 0.74; 0.09, 1.39)***** - Bed sharing: **(OR: 0.53; 0.24, 0.82)*** - Personal toilet: **(OR: 0.73; 0.59, 0.88)*** - Hot water: **(OR: 0.76; 0.57, 0.95)*** - Greater number of siblings: (OR: 0.98; 0.94, 1.3)***** |
| Frias Gomes C et al.^3^ | 2021 | Cesarean delivery | 10 | IBD risk: (OR: 1.01; 0.81, 1.27; *I*^2^=87%)  CD risk: (OR: 1.15; 0.94, 1.42; *I*^2^=55%)  UC risk: (OR: 0.94; 0.61, 1.45; *I*^2^=55%) |
| Bruce A et al.^4^ | 2014 | Cesarean delivery | 7 | IBD risk: (OR: 1.00; 0.75, 1.33; *I*^2^=90%) |
| Li Y et al.^5^ | 2014 | Cesarean delivery | 6 | IBD risk: (OR: 1.13; 0.99, 1.30; *I*^2^=63%)  CD risk: **(OR: 1.38; 1.12, 1.70; *I*^2^=61%)**  UC risk: (OR: 1.07; 0.87, 1.32; *I*^2^=0%) |
| Xu L et al.^6^ | 2017 | Breastfeeding | 35 | IBD risk: **(OR: 0.74; 0.66, 0.83; *I*^2=^72%)**  CD risk: **(OR: 0.71; 0.59, 0.85; *I*^2^=78%)**  UC risk: **(OR: 0.78; 0.67, 0.91; *I*^2^=63%)**  Dose-dependent association >12 months compared to 3–6 months:   - CD: **(OR: 0.20; 0.08, 0.50)*** - UC: **(OR: 0.21; 0.10, 0.43)*** |
| Klement E et al.^7^ | 2004 | Breastfeeding | 17 | CD risk: **(OR: 0.67; 0.52, 0.86)***  UC risk: **(OR: 0.77; 0.61, 0.96)*** |
| **DIET** | | | | |
| Thacker N et al.^8^ | 2024 | Antibiotics, passive smoking, high socioeconomic status and sweetened foods | 3  3  4  2 | Breastfed   - Pediatric IBD: (OR 1.62; 0.53, 5; *I*^2^=88.06%) - Pediatric CD: (OR 0.40; 0.11, 1.05; *I*^2^=84.53%) - Pediatric UC: (OR 0.70; 0.45, 1.09; *I*^2^=49.07%)   Sugary beverage   - **Pediatric IBD: (OR 2.02; 1.06, 3.85; *I*^2^=0%)**   Vegetable intake   - Pediatric IBD: (OR 0.47; 0.21, 1.06; *I*^2^=52.70%) |
| Almofarreh AM et al.^9^ | 2024 | Dairy consumption | 19 | UC risk: (OR 0.82; 0.68, 0.98 *I*^2^=47.62%)  CD risk: (OR0.72; 0.59, 0.87 *I*^2^=41.16%) |
| Salavatizadeh M et al.^10^ | 2022 | Micronutrient intake | 7 | UC risk:   - Intake of calcium (WMD: -66.25mg/d; -276.7- 144.21, *I*^2^=95.1%) - Intake of magnesium (WMD: -21.47mg/d; -95.4- 52.6, *I*^2^=96%) - Intake of zinc (WMD: 0.3mg/d; -1.5- 2, *I*^2^=95.8%) |
| Talebi S et al.^11^ | 2023 | Total protein, Animal protein and Animal protein sources | 11 | IBD risk:   - **Dairy: (RR: 0.81; 0.72, 0.90; *I^2^*=36.2%)** - Animal protein: (RR: 1.23; 0.81, 1.86; *I^2^*=62.5%) - Red meat: (RR: 1.10; 0.97, 1.25; *I^2^*=55.6%) - Processed meat: (RR: 1.09; 0.94, 1.26; *I^2^*=61.04%) - Poultry: (RR: 1.18; 0.88, 1.59; *I^2^*=45.4%) - Fish: (RR: 1.03; 0.92, 1.15; *I^2^*=25.4%) - Egg: (RR: 0.92; 0.81, 1.04; *I^2^*=0%)   Dose-response meta-analysis:   - **Total meat dietary increment 100g/d: (RR: 1.38; 1.13, 1.68)*** - Animal protein dietary increment 100g/d: (RR: 0.98; 0.59, 1.64; *I^2^*=0%) - Red meat dietary increment 100g/d: (RR: 1.34; 0.96, 1.86; *I^2^*=33.4%) - Poultry dietary increment 100g/d: (RR: 1.74; 0.49, 6.13; *I^2^*=51.9%) - Fish dietary increment 15g/d: (RR: 0.99; 0.88, 1.11; *I^2^*=49.8%) - Dairy dietary increment 200g/d: (RR: 0.97; 0.92, 1.01; *I^2^*=0%) - Egg dietary increment 50g/d: (RR: 0.99; 0.65, 1.52; *I^2^*=0%)   CD risk   - **Dairy: (RR: 0.69; 0.56, 0.86)** - Animal protein: (RR: 1.34; 0.86, 2.10; *I^2^*=4.1%) - Red meat: (RR: 1.02; 0.82, 1.28; *I^2^*=66.5%) - Processed meat: (RR: 1.01; 0.78, 1.30; *I^2^*=65.9%) - Poultry: (RR: 1.42; 0.87, 2.33)***** - Fish: (RR: 0.92; 0.73, 1.15, *I^2^*=43.4%) - Egg: (RR 0.94; 0.78, 1.12, *I^2^*=0%)   UC risk:   - **Dairy: (RR: 0.84; 0.75, 0.94; *I^2^*=0%)** - Animal protein (RR: 1.20; 0.63, 2.30; *I^2^*=74.2%) - Red meat: (RR: 1.16; 0.96, 1.40; *I^2^*=59%) - Processed meat: (RR: 1.16; 0.99, 1.37; *I^2^*=32%) - Poultry: (RR: 0.92; 0.67, 1.26)***** - Fish: (RR: 1.07; 0.95, 1.20; *I^2^*=0%) - Egg: (RR: 0.90; 0.65, 1.52; *I^2^*=0%) |
| Zhou XL et al.^12^ | 2022 | Protein intake | 12 | IBD risk: cohort studies (RR: 1.56; 0.38, 6.25; *I^2^*=86.4%)  Case-control (RR: 1.06; 0.66, 1.69; *I^2^*=49%) |
| Ge J et al^13^ | 2015 | Meat intake | 9 | IBD risk: **(RR: 1.50; 1.15, 1.95)*** |
| Mozaffari H et al.^14^ | 2020 | Fish, n-3 PUFA | 12 | IBD risk:   - Fish: (Effect Size: 0.68; 0.46, 1; *I^2^*=76.4%) - N3 PUFA: (Effect Size: 1.17; 0.80, 1.72; *I^2^*=57.3%)   CD risk:   - **Fish: (Effect Size: 0.54; 0.31, 0.96,  *I^2^*=80.5%)** - N3 PUFA: (Effect Size: 1.62; 0.38, 6.87; *I^2^*=0%)   UC risk   - Fish: (Effect Size: 0.82; 0.56, 1.22,  *I^2^*=30.6%) - N3 PUFA: (Effect Size 0.96; 0.75, 1.22,  *I^2^*=0%**)** |
| Wang F et al.^15^ | 2017 | Fat intake | 9 | UC risk:   - Total fat: (RR: 1.02; 0.96, 1.09; *I^2^*=24%) - Saturated fat: (RR: 1.06; 0.85, 1.34; *I^2^*=44.5%) - MUFA: (RR: 1.21; 0.91, 1.62; *I^2^*=63.1%) - PUFA: (RR: 1.25; 0.95, 1.64; *I^2^*=25%) |

| Milajerdi A et al.^16^ | 2021 | Dietary Fiber, Fruit & Vegetable | 6 | CD risk:   - **Dietary fiber: (RR: 0.59; 0.46, 0.74, *I^2^*=0%)** - **Fruit: (RR: 0.47; 0.38, 0.58, *I^2^*=32.1%)** - **Vegetables: (RR: 0.52; 0.46, 0.59,  *I^2^*=78.9%)**   UC risk:   - Dietary fiber: (RR: 1.09; 0.88, 1.34, ***I^2^*=0%**) - **Fruit: (RR: 0.69; 0.55, 0.86, *I^2^*=87%)** - **Vegetables: (RR: 0.56; 0.48, 0.66,  *I^2^*=72%)** |
| --- | --- | --- | --- | --- |
| Liu X et al.^17^ | 2015 | Dietary fiber | 8 | CD risk: **(RR: 0.44; 0.29, 0.69, *I^2^*=56.1%)**  UC risk: (RR: 0.80; 0.64, 1.00, *I^2^*=48%) |
| Li F et al.^18^ | 2015 | Vegetables and fruit | 14 | Vegetables   - CD risk: (OR: 0.66; 0.40, 1.09, *I^2^*=67.5%) - UC risk: **(OR: 0.71; 0.58, 0.88,  *I^2^*=41.6)**   Fruits   - CD risk: **(OR: 0.57; 0.44, 0.74, *I^2^*=59.5%)** - UC risk: **(OR: 0.69; 0.49, 0.96, *I^2^*=50.7)** |
| Jin ZQ et al.^19^ | 2019 | Carbohydrate intake | 15 | IBD risk: (OR: 1.09; 0.82, 1.46; *I^2^*=31.6%)  CD risk: (OR:1.01; 0.63, 1.62,  *I^2^*=18.4%)  UC risk: (OR:1.17; 0.78, 1.75,  *I^2^*=44.4) |
| Wang F et al.^20^ | 2017 | Carbohydrate & protein | 9 | UC risk:   - Carbohydrate: (RR: 1.00; 099, 1.02; *I^2^*=31.5%) - Protein: (RR: 1.01; 0.98, 1.05; *I^2^*=12.4%) - Fiber intake: (RR: 1.00; 0.97, 1.03; *I^2^*=0%) - Sugar: (RR: 1.03; 0.96, 1.10; *I^2^*=68.9%) - **Sucrose: (RR: 1.10; 1.02, 1.18; *I^2^*=0%)** |
| Zeng L et al.^21^ | 2017 | Macronutrient intake | 9 | CD risk:   - Carbohydrate: (RR: 0.99; 0.98, 1.00, *I^2^*=54.7%) - Fat: (RR: 1.02; 0.97, 1.07, *I^2^*=44.6%) - Protein: (RR: 1.03; 0.96, 1.11, *I^2^*=54.7%) - **Fiber: (RR: 0.85; 0.76, 0.96, *I^2^*=0%)** - **Sucrose: (RR: 1.09; 1.02, 1.16,  *I^2^*=0%)** |
| Yang Y et al.^22^ | 2019 | Beverage consumption | 16 | CD risk:   - Alcohol: (RR: 0.85; 0.68, 1.08, *I^2^*=0) - Coffee intake: (RR: 0.82; 0.46, 1.46, *I^2^*=81%) - **Soft drinks: (RR: 1.43; 1.01, 1.98, *I^2^*=78.1%)** - **Tea: (RR: 0.70; 0.53, 0.93, *I^2^*=0%)** |
| Nie JY et al.^23^ | 2017 | Beverage consumption | 16 | UC risk:   - Alcohol: (RR: 0.95; 0.65, 1.39, *I^2^*=66%) - Coffee: (RR: 0.58; 0.33, 1.05, *I^2^*=87.5%) - **Soft drinks: (RR: 1.69; 1.34, 2.30, *I^2^*=12.9%)** - **Tea: (RR: 0.69; 0.58, 0.83, *I^2^*=0%)** |
| Li T et al.^24^ | 2020 | Western Diet | 9 | **IBD risk: (RR: 1.92; 1.37, 2.68 *I^2^*=70.4%)**  **CD risk: (RR: 1.72; 1.01, 2.93, *I^2^*=74.8%)**  **UC risk: (RR: 2.15; 1.38, 3.34, *I^2^*=65.9%)** |
| Narula N et al.^25^ | 2023 | Ultra-processed food | 5 | CD risk: **(HR: 1.71; 1.37, 2.14; *I^2^*=0%)**  UC risk: (HR: 1.17;0.86, 1.61; *I^2^*=74%) |
| Babaei A et al.^26^ | 2023 | Ultra-processed food | 24 | IBD risk: (**RR: 1.13; 1.06, 1.21; *I^2^*=73.2%**)  CD risk: **(RR: 1.19; 1.00, 1.41; *I^2^*=78.2%)**  UC risk: (RR: 1.11; 0.99, 1.26; *I^2^*=60.3%) |
| **SMOKING** | | | | |
| Thacker N et al.^8^ | 2024 | Antibiotics, passive smoking, high socioeconomic status and sweetened foods | 3  4 | Passive smoking   - **Pediatric IBD: (OR 2.39; 1.50, 3.80, *I^2^*=0%)**   Maternal smoking & CD   - **Pediatric CD: (OR 1.64; 1.14, 2.36, *I^2^*=8.58%)** |
| Larsson SC et al.^27^ | 2022 | Genetic liability to smoking | 6 | CD: **(OR: 1.15; 1.01, 1.32)***  UC: (OR: 1.02; 0.92, 1.14)***** |
| Piovani D et al.^28^ | 2021 | Ethnic differences in smoking risk | 57  29  11  51 | CD risk:   - Non-Jewish, white smokers: **(RR: 1.95; 1.69, 2.24, *I^2^*=83.8%)** - Asian, Jewish, and Latin-American (RR: 0.97; 0.83, 1.13, *I^2^*=85.4%)   UC risk:  Current smokers: **(RR: 0.55; 0.48, 0.64, *I^2^*=85.7%) irrespectively of ethnicity** |
| Jones DT et al.^29^ | 2008 | Passive smoking during childhood | 13 | CD risk:   - Childhood passive smoking: (OR: 1.10; 0.92, 1.30, *I^2^*=45.9%) - Prenatal smoker exposure: (OR: 1.10; 0.67, 1.80, *I^2^*=75.1%)   UC risk:   - Childhood passive smoking: (OR: 1.01; 0.85, 1.20, *I^2^*=22.1%) - Prenatal smoker exposure: (OR: 1.11; 0.63, 1.97, *I^2^*=72.1%) |
| Mahid SS et al.^30^ | 2006 | Smoking | 9  13 | CD risk:   - Current smoker: **(OR: 1.76; 1.40, 2, 22, p<0.001)*** - Former smoker: (OR: 1.30; 0.97, 1.76, p=0.08)*****   UC risk:   - Current smoker: **(OR: 0.58; 0.45, 0.75; p<0.001)*** - Former smoker: **(OR: 1.79; 1.37, 2.34, p<0.001)*** - Ever smoker (current/former): (OR: 0.98; 0.81, 1.17, p=0.79)***** |
| Labarca G et al.^31^ | 2019 | Chronic obstructive pulmonary disease | 4  (only 1 reported tobacco exposure) | IBD risk: **(RR: 2.02; 1.56, 2.63; *I*^2^=72%)**  CD risk: **(RR: 2.29; 1.51, 3.48; *I*^2^=62%)**  UC risk: **(RR: 1.79; 1.39, 2.29; *I*^2^=19%)** |
| **PHYSICAL ACTIVITY & WEIGHT** | | | | |
| Tiong HT et al.^32^ | 2024 | Physical activity | 10 | CD risk:   - **Cohort studies: (OR: 0.78; 0.68, 0.88; *I*^2^=0%)** - **Case-control: (OR: 0.87; 0.79, 0.95; *I*^2^=69%)**   UC risk:   - **Cohort studies: (OR: 0.62; 0.43, 0.88; *I*^2^=0%)** - Case control: (OR: 0.74; 0.51, 1.07; *I*^2^=81%) |
| Wang Q et al.^33^ | 2016 | High physical activity | 6 | CD risk: **(RR: 0.63; 0.50, 0.79; *I*^2^=3%)**  UC risk: (RR: 0.82; 0.68, 1.00; *I*^2^=0%) |
| Chan SSM et al.^34^ | 2022 | Obesity | 5 | CD risk: **(aHR: 1.34; 1.05, 1.71; *I*^2^=0%)** |
| Milajerdi A et al.^35^ | 2022 | Obesity | 9 | CD risk: (RR: 0.87; 0.73, 1.05; *I^2^*=88.9%)  UC risk: (RR: 0.79; 0.68, 0.92; *I^2^*=0.0%) |
| Rahmani J et al.^36^ | 2019 | Obesity | 5 | CD risk: **(HR: 1.42; 1.18, 1.71; *I*^2^=0%)**  UC risk: (HR: 0.96; 0.80, 1.14; *I*^2^=8%) |
| Dong J et al.^37^ | 2015 | Body Mass Index | 24 | CD: (weighted mean difference [WMD]: −1.88; −2.77, −1.00; *p*<0.001)*****  UC: (WMD: −0.94; −2.54, 0.66; *p*<0.001)*****  IBDu: (WMD: −2.64; −5.43, 0.16; *p*=0.064)***** |
| **MEDICATIONS** | | | | |
| Wang X et al.^38^ | 2019 | Oral contraceptives | 12 | UC risk:   - **Oral contraceptive exposure: (OR: 1.22; 1.06, 1.41; *I^2^*=30%)** - **Current oral contraceptive user (OR: 1.49; 1.12, 1.96; *I^2^*=3.2%)** - Past oral contraceptive use (OR: 1.17; 0.95, 1.43; *I^2^*=0%). |
| Ortizo R et al.^39^ | 2017 | Oral Contraceptives | 20 | IBD risk: **(OR: 1.32; 1.17, 1.49; *I^2^*=14%)**  CD risk: **(OR: 1.24; 1.09, 1.40; *I^2^*=38%)**  UC risk: **(OR: 1.30; 1.13, 1.49; *I^2^*=26%)** |
| Cornish JA et al.^40^ | 2008 | Oral Contraceptives | 14 | CD risk: **(RR: 1.51; 1.17, 1.96)**; **(RR: 1.46; 1.26, 1.70)*** adjusted for smoking  UC risk: **(RR: 1.53; 1.21, 1.94);** **(RR: 1.28; 1.06, 1.54)*** adjusted for smoking |
| Godet PG et al.^41^ | 1995 | Oral contraceptives | 9 | CD risk (adjusted for smoking): (**OR: 1.44; 1.12, 1.86**)*****  UC risk (adjusted for smoking): (OR: 1.29; 0.94, 1.77)***** |
| Yu CL et al.^42^ | 2023 | Isotretinoin | 8 | IBD risk: (OR: 1.01; 0.80, 1.27)*****  CD risk: (OR: 0.87; 0.65, 1.15)*****  UC risk: (OR: 1.27; 0.94, 1.73)***** |
| Lee SE et al.^43^ | 2016 | Isotretinoin | 6 | IBD risk: (OR: 1.08; 0.82, 1.42, *I^2^*=68%)  CD risk: (OR: 0.98; 0.62, 1.55; *I^2^*=62%)  UC risk: (OR: 1.14; 0.79, 1.63; *I^2^*=44%) |
| Etminan M et al.^44^ | 2013 | Isotretinoin | 5 | IBD risk: (RR: 0.94; 0.65, 1.36, *I^2^*=67%)  CD risk: (RR 0.75; 0.46,1.24, *I^2^*=40%)  UC risk: (RR 1.61; 0.88, 2.95, *I^2^*=63%) |
| Shastri SA et al.^45^ | 2022 | Proton-Pump inhibitors | 8 | IBD risk: **(aOR: 2.43; 1.18, 5.02; *I^2^*=86%)**  UC AND CD risk: (aOR 3.60; 1.10, 11.74)*  UC risk: (aOR: 1.50; 0.50, 4.50)* |
| Thacker N et al.^8^ | 2024 | Antibiotics, passive smoking, high socioeconomic status and sweetened foods | 3  2 | Any antibiotic exposure   - **Pediatric IBD: (OR 4.82; 2.45, 9.48)** - **Pediatric CD: (OR 2.08; 1.32, 3.29)**   Early childhood antibiotic exposure   - **Pediatric CD: (OR 2.46; 1.29, 4.67, *I^2^*=0%)**   Exposure to >4 courses of antibiotics   - **Pediatric IBD: (OR 3.64; 1.45, 14.77)** - **Pediatric CD: (OR 4.73; 2.40, 8.95)** |
| Zhu JK et al.^46^ | 2024 | Antibiotic exposure | 16 | UC risk: (OR 1.28; 1.26, 1.31) |
| Duan R et al.^47^ | 2024 | Antibiotics | 28 | IBD risk:   - Prescription based studies (**pOR: 1.41; 1.29, 1.53; *I^2^*=95.3%)** - Questionary based studies (**pOR: 1.35; 1.08, 1.68; *I^2^*=82.1%)**   CD risk:   - Prescription based studies (**pOR: 1.47; 1.34, 1.62, *I^2^*=83.9%)** - Questionary based studies (**pOR: 1.45; 1.10, 1.92, *I^2^*=88.4%)**   UC risk:   - Prescription based studies (**pOR: 1.30; 1.20, 1.40; *I^2^*=95.3%)** - Questionary based studies (pOR: 1.27; 0.89, 1.8, *I^2^*=84%) |
| Dar SH et al.^48^ | 2023 | Antibiotics | 12 | CD risk: (**OR: 1.52; 1.23, 1.87)***  UC risk: (OR: 1.11; 0.93, 1.33)***** |
| Zou Y et al.^49^ | 2020 | Antibiotics during childhood | 11 | IBD risk: **(OR: 2.00; 1.05, 3.83; *I^2^*=81.2%)**  CD risk: **(OR: 1.59; 1.06, 2.4; *I^2^*=83.6%)**  UC risk: (OR: 1.22; 0.82, 1.8; *I^2^*=70.9%) |
| Ungaro R et al.^50^ | 2014 | Antibiotics | 11 | IBD risk:   - Any antibiotic: **(OR: 1.57; 1.27, 1.94; *I^2^*=92.9 %)** - Metronidazole: **(OR: 5.01; 1.65, 15.25; *I^2^*=91%)** - Fluoroquinolones: **(OR: 1.79; 1.03, 3.12; *I^2^*=85.9 %)** - Broad-spectrum penicillins: (OR: 1.31; 1.14, 1.51; ***I^2^*=70.8%)** - Tetracyclines: **(OR: 1.30; 1.17, 1.44; *I^2^*=0%)** - Cephalosporins: **(OR: 1.27; 1.14, 1.41; *I^2^*=6.7%)** - Macrolides: **(OR: 1.23; 1.11, 1.36; *I^2^*=20%)** - Sulfonamides: **(OR: 1.17; 1.06, 1.30; *I^2^*=0%)** - Penicillin: (OR: 1.12; 0.76, 1.64; *I^2^*=74.2 %)   CD risk: **(OR: 1.74; 1.35, 2.23; *I^2^*=84.9%)**  UC risk: (OR: 1.08; 0.91, 1.27; *I^2^*=47.4%)  Pediatric CD: **(OR: 2.75; 1.72, 4.38, *I^2^*=0%)** |
| **INFECTIONS** | | | | |
| Kong G et al.^51^ | 2023 | Helicobacter pylori | 6 | Pediatric IBD risk: (OR: 0.62; 0.34, 1.12; *I^2^*=35%) |
| Zhong Y et al.^52^ | 2021 | Helicobacter pylori | 23 | IBD risk: (OR: 0.44; 0.34, 0.59)*****  CD risk: (OR: 0.36; 0.26, 0.49)*****  UC risk: (OR: 0.54, 0.4, 0.72)***** |
| Shirzad-Aski H et al.^53^ | 2021 | Helicobacter pylori | 58 | IBD (pOR: 0.45; 0.39, 0.53; *I^2^*=79%) |
| Tepler A et al.^54^ | 2019 | Helicobacter pylori cagA vs Helicobacter cagA-*  Helicobacter pylori cagA+ vs No Helicobcter  Helicobacter pylori cagA- vs No Helicobcter | 3 | IBD risk: **(OR: 0.31; 0.21, 0.44; *I^2^*=0%)***  CD risk: **(OR: 0.25; 0.17, 0.38; *I^2^*=0%)***  UC risk: (OR: 0.68; 0.35, 1.32; *I^2^*=0%)*  IBD risk: (**OR: 0.25; 0.16, 0.41; *I^2^*=35%)**  CD risk: (OR: 0.23; 0.15, 0.38; *I^2^*=0%)  UC risk: (OR: 0.66; 0.34, 1.27; *I^2^*=0%)  IBD risk: **(**OR: 0.74; 0.46, 1.17; *I^2^*=88%)  CD risk: (OR: 0.97; 0.82, 1.14; *I^2^*=0%)  UC risk: (OR: 0.97; 0.76, 1.24; *I^2^*=0%) |
| Wang WL et al.^55^ | 2019 | Helicobacter pylori | 20 | CD risk: **(OR: 0.42; 0.33, 0.54; *I^2^*=64%)** |
| Castaño-Rodríguez N et al.^56^ | 2017 | Helicobacter pylori  Campylobacter spp  Enterohepatic Helicobacter spp (EHS) | 40  9  15 | IBD risk:   - Helicobacter pylori: **(OR: 0.43; 0.36, 0.50; *I^2^*=61.81%)** - Campylobacter spp: **(OR: 2.97; 1.33, 6.63; *I^2^*=82.3%)** - EHS: (OR: 1.51; 0.95, 2.41; *I^2^*=57.3%)   CD risk:   - Helicobacter pylori: **(OR: 0.38; 0.31, 0.47)***   UC risk:   - Helicobacter pylori (**OR: 0.53; 0.44, 0.65)***   IBDU risk:   - Helicobacter pylori **(OR: 0.43; 0.23, 0.80)*** |
| Wu XW et al.^57^ | 2015 | Helicobacter pylori in Asians | 10 | IBD risk: (RR: 0.48; 0.43, 0.54; *I^2^*=21%) |
| Yu Q et al.^58^ | 2015 | Enterohepatic Helicobacter spp  Intestinal Helicobacter pylori | 14 | IBD risk:   - **EHS: (RR: 2.01; 1.36, 2.98; *I^2^*=15.9%)** - Intestinal HP: (RR: 1.22; 0.77, 1.95; *I^2^*=59.9%)   CD risk:   - **EHS: (RR: 1.72; 1.20, 2.47; *I^2^*=0%)** - Intestinal HP: (RR: 1.30; 0.74, 2.27; *I^2^*=57.5%)   UC risk:   - EHS: (RR: 3.27; 0.93, 11.44; *I^2^*=51%) - Intestinal HP: (RR: 1.13; 0.44, 2.90; *I^2^*=68.7%) |
| Rokkas T et al.^59^ | 2015 | Helicobacter pylori | 33 | IBD risk: **(RR: 0.62; 0.55, 0.71; *I^2^*=77%)** |
| Luther J et al.^60^ | 2010 | Helicobacter pylori | 23 | IBD risk: (RR: 0.69; 0.54, 0.75; *I^2^*=75.8%) |
| Pineton de Chambrun G et al.^61^ | 2015 | Vaccination against:   - Bacille Calmette-Guérin (BCG) - Diphteria - Smallpox - Poliomyelitis - Measles - H1N1 | 11 | IBD risk:   - BCG: (RR: 1.04; 0.78, 1.38; *I^2^*=62%) - Diphteria: (RR: 1, 24; 0.80; 1.94, *I^2^*<50%) - Smallpox: (RR: 1.08; 0.70, 1.27; *I^2^*<50%) - Poliomyelitis: **(RR: 1.79; 0.88, 3.66; *I^2^*=67%)** - Measles: (RR: 1.33; 0.31, 5.80; *I^2^*=74%) - H1N1: (RR: 1.31; 0.97, 1.32)***** |
| Huang W et al.^62^ | 2024 | HIV | 7 | IBD risk: (OR 2.68; 1.17, 6.13 *I^2^*=99.9%) |
| **SURGERY** | | | | |
| Amin R et al.^1^ | 2024 | Appendicectomy  Tonsillectomy | 7 | **CD risk: (OR 1.57; 1.01, 2.43, *I^2^*=93%)**  UC risk: (OR 0.60; 0.24, 1.47, *I^2^*=96%)  CD risk: (OR 1.93; 0.96, 3.89, *I^2^*=62%)  **UC risk: (OR 1.24; 1.18, 1.30, *I^2^*=0%)** |
| Zhang L et al.^63^ | 2023 | Appendicectomy | 28 | CD risk:   - Case controls: (OR: 1.59; 1.22, 2.08)***** - Cohort studies: (RR: 2.28; 1.66, 3.14)***** |
| Deng P et al.^64^ | 2016 | Appendicectomy | 16 | UC risk: (OR: 0.44; 0.30, 0.64, *I^2^*=82%) |
| Kaplan GG et al.^65^ | 2008 | Appendicectomy | 21 | CD: **(RR: 1.61; 1.28, 2.02; *p*<0.001)*** |
| Koutroubakis IE et al.^66^ | 2000 | Appendicectomy | 13 | UC risk: **(OR: 0.307; 0.249, 0.377; *I*^2^=16.2%)** |
| Kermansaravi M et al.^67^ | 2022 | Bariatric surgery | 4 | IBD risk: **(OR: 1.17; 1.06, 1.29; *I*^2^=90.2%)** |
| Sun W et al.^68^ | 2016 | Tonsillectomy | 17  22 | CD risk: **(OR: 1.37; 1.16, 1.62; *I*^2^=3%)**   - **Adjusted for smoking: (OR: 1.66; 1.03, 2.68)***   UC risk: (OR: 0.94; 0.84, 1.05; *I*^2^=0%)   - Adjusted for smoking: (OR: 1.03; 0.74, 1.44)***** |
| Xiong HF et al.^69^ | 2016 | Tonsillectomy | 13 | CD risk: (OR: 1.194; 0.992, 1.437)*****  UC risk: (OR: 0.932; 0.819, 1.062)***** |
| **LATITUDE & VITAMIN D** | | | | |
| Holmes EA et al.^70^ | 2015 | Latitude & ultraviolet radiation in pediatric IBD | 39 | CD risk:   - Latitude (10º): **(OR: 0.23; 0.02, 0.44; *p*=0.03; R^2^=2.9%)** - Hemisphere (South vs North): (OR: 0.52; −0.23, 1.26, R^2^=2.9%) - Ambient ultraviolet radiation: (OR: −0.19; −0.45, 0.07, R^2^=1.5%) |
| Lu C et al.^71^ | 2015 | Latitude & ultraviolet radiation and geographical location | 13 | CD risk:   - Vitamin D deficiency (OR: 1.95; 1.48, 2.57)*****   UC risk:   - Vitamin D deficiency (OR: 2.02; 1.13, 3.60)***** |
| Del Pinto R et al.^72^ | 2015 | Vitamin D levels | 14 | IBD risk: **(OR: 1.64; 1.30, 2.08; *I*^2^=7%)**  CD risk: **(OR: 1.63; 1.24, 2.13; *I*^2^=0%)**  UC risk: **(OR: 2.28; 1.18, 4.41; *I*^2^=41%)** |
| Wang L et al.^73^ | 2014 | Vitamin D receptor gene polymorphisms (GP) | 6  5  5  8 | IBD:   - GP ApaI: (OR: 1.16; 0.92, 1.46, *I*^2^=0%) - GP BsmI: (OR: 1.11; 0.96, 1.29, *I*^2^=33.7%) - GP FokI: (OR: 0.87; 0.71, 1.07, *I*^2^=76.9%) - GP Taql: **(OR: 0.90; 0.83, 0.98; *I*^2^=0%)**   CD:   - GP ApaI (AA vs aa): **(OR: 1.40; 1.05, 1.88, *I*^2^=0%)** - GP BsmI (East Asians): **(OR: 1.77; 1.14, 2.74)*** - GP Taql (Caucasians): (OR: 0.79; 0.63, 1.00, *I*^2^=0%)   UC:   - GP Taql (Caucasians): **(OR: 0.89; 0.80, 0.99, *I*^2^=0%)** |
| Xue LN et al.^74^ | 2013 | Vitamin D receptor gene polymorphisms | 9 | CD risk   - GP ApaI: **(OR: 0.81; 0.67, 0.97)*** - GP FokI: (OR: 1.02; 0.87, 1.20)***** - GP BsmI: (OR: 1.17; 0.93, 1.48)***** - GP TaqI: (OR: 1.19; 0.99, 1.43)***** - GP TaqI (in Europeans): **(OR: 1.23; 1.02, 1.49)*** - GP TaqI (in males): **(OR: 1.84; 1.19, 2.83)***   UC risk:   - GP ApaI: (OR: 1.07; 0.90, 1.26)***** - GP BsmI: (OR 1.16; 0.90, 1.49)***** - GP FokI: (OR: 1.01; 0.87, 1.18)***** - GP FokI (in Asians) **(OR: 1.65; 1.11, 2.45)*** - GP TaqI (in males): **(OR: 1.56; 1.02, 2.39)*** |
| Liu C et al.^75^ | 2023 | Vitamin D levels | 16 | **UC risk: (OR: 1.90; 1.38, 2.62; *I*^2^=92.7%)** |
| **URBAN VERSES RURAL LIVING** | | | | |
| Song C et al.^76^ | 2019 | Urban vs rural during childhood | 15 | IBD risk: **(OR: 1.34; 1.11, 1.62; *I*^2^=71.2%)**  CD risk: **(OR: 1.45; 1.14, 1.85; *I*^2^=73.9%)**  UC risk: (OR: 1.16; 0.83, 1.61; *I*^2^=75.2%) |
| Soon IS et al.^77^ | 2012 | Urban environment vs rural | 30 CD  25 UC | CD risk: **(IRR: 1.42; 1.26, 1.60; *I*^2^=76%)**  UC risk: **(IRR: 1.17; 1.03, 1.32; *I*^2^=79.1%)** |

*I^2^ was not provided by source meta-analysis

**References for the Supplementary File**

1. Agrawal M, Sabino J, Frias-Gomes C, et al. Early life exposures and the risk of inflammatory bowel disease: Systematic review and meta-analyses. *EClinicalMedicine*. Jun 2021;36:100884. doi:10.1016/j.eclinm.2021.100884

2. Cholapranee A, Ananthakrishnan AN. Environmental Hygiene and Risk of Inflammatory Bowel Diseases: A Systematic Review and Meta-analysis. *Inflamm Bowel Dis*. Sep 2016;22(9):2191-9. doi:10.1097/MIB.0000000000000852

3. Frias Gomes C, Narula N, Morão B, Nicola P, Cravo M, Torres J. Mode of Delivery Does Not Affect the Risk of Inflammatory Bowel Disease. *Digestive diseases and sciences*. Feb 2021;66(2):398-407. doi:10.1007/s10620-020-06204-7

4. Bruce A, Black M, Bhattacharya S. Mode of delivery and risk of inflammatory bowel disease in the offspring: systematic review and meta-analysis of observational studies. *Inflamm Bowel Dis*. Jul 2014;20(7):1217-26. doi:10.1097/MIB.0000000000000075

5. Li Y, Tian Y, Zhu W, et al. Cesarean delivery and risk of inflammatory bowel disease: a systematic review and meta-analysis. *Scand J Gastroenterol*. Jul 2014;49(7):834-44. doi:10.3109/00365521.2014.910834

6. Xu L, Lochhead P, Ko Y, Claggett B, Leong RW, Ananthakrishnan AN. Systematic review with meta-analysis: breastfeeding and the risk of Crohn's disease and ulcerative colitis. *Aliment Pharmacol Ther*. 2017;46(9):780-789. doi:<https://doi.org/10.1111/apt.14291>

7. Klement E, Cohen RV, Boxman J, Joseph A, Reif S. Breastfeeding and risk of inflammatory bowel disease: a systematic review with meta-analysis. *Am J Clin Nutr*. Nov 2004;80(5):1342-52.

8. Thacker N, Duncanson K, Eslick GD, et al. Antibiotics, passive smoking, high socioeconomic status and sweetened foods contribute to the risk of paediatric inflammatory bowel disease: A systematic review with meta-analysis. *Journal of Pediatric Gastroenterology and Nutrition*. 2024;79(3):610-621. doi:<https://doi.org/10.1002/jpn3.12303>

9. Almofarreh AM, Sheerah HA, Arafa A, et al. Dairy Consumption and Inflammatory Bowel Disease among Arab Adults: A Case–Control Study and Meta-Analysis. *Nutrients*. 2024;16(16):2747.

10. Salavatizadeh M, Soltanieh S, Chegini M, Ilesanmi-Oyelere BL, Kord-Varkaneh H, Hekmatdoost A. Micronutrient intake and risk of ulcerative colitis: A meta-analysis of observational studies. *Clin Nutr ESPEN*. Oct 2022;51:152-159. doi:10.1016/j.clnesp.2022.07.008

11. Talebi S, Zeraattalab-Motlagh S, Rahimlou M, et al. The Association between Total Protein, Animal Protein, and Animal Protein Sources with Risk of Inflammatory Bowel Diseases: A Systematic Review and Meta-Analysis of Cohort Studies. *Adv Nutr*. Jul 2023;14(4):752-761. doi:10.1016/j.advnut.2023.05.008

12. Zhou XL, Zhao QQ, Li XF, Li Z, Zhao SX, Li YM. Protein intake and risk of inflammatory bowel disease: A meta-analysis. *Asia Pac J Clin Nutr*. 2022;31(3):443-449. doi:10.6133/apjcn.202209_31(3).0012

13. Ge J, Han TJ, Liu J, et al. Meat intake and risk of inflammatory bowel disease: A meta-analysis. *The Turkish journal of gastroenterology : the official journal of Turkish Society of Gastroenterology*. 2015/11// 2015;26(6):492-497. doi:10.5152/tjg.2015.0106

14. Mozaffari H, Daneshzad E, Larijani B, Bellissimo N, Azadbakht L. Dietary intake of fish, n-3 polyunsaturated fatty acids, and risk of inflammatory bowel disease: a systematic review and meta-analysis of observational studies. *Eur J Nutr*. 2020/02/01 2020;59(1):1-17. doi:10.1007/s00394-019-01901-0

15. Wang F, Lin X, Zhao Q, Li J. Fat intake and risk of ulcerative colitis: Systematic review and dose–response meta-analysis of epidemiological studies. *J Gastroenterol Hepatol*. 2017/01/01 2017;32(1):19-27. doi:<https://doi.org/10.1111/jgh.13416>

16. Milajerdi A, Ebrahimi-Daryani N, Dieleman LA, Larijani B, Esmaillzadeh A. Association of Dietary Fiber, Fruit, and Vegetable Consumption with Risk of Inflammatory Bowel Disease: A Systematic Review and Meta-Analysis. *Adv Nutr*. Jun 1 2021;12(3):735-743. doi:10.1093/advances/nmaa145

17. Liu X, Wu Y, Li F, Zhang D. Dietary fiber intake reduces risk of inflammatory bowel disease: result from a meta-analysis. *Nutr Res*. 2015/09/01/ 2015;35(9):753-758. doi:<https://doi.org/10.1016/j.nutres.2015.05.021>

18. Li F, Liu X, Wang W, Zhang D. Consumption of vegetables and fruit and the risk of inflammatory bowel disease: a meta-analysis. *Eur J Gastroenterol Hepatol*. Jun 2015;27(6):623-30. doi:10.1097/meg.0000000000000330

19. Jin Z-Q, Lu H-G, Wu Q-B, et al. A meta-analysis of dietary carbohydrate intake and inflammatory bowel disease risk: evidence from 15 epidemiology studies. *Rev Esp Enferm Dig*. 2019;111(1):5-9. doi:10.17235/reed.2018.5490/2018

20. Wang F, Feng J, Gao Q, et al. Carbohydrate and protein intake and risk of ulcerative colitis: Systematic review and dose-response meta-analysis of epidemiological studies. *Clinical Nutrition*. 2017/10/01/ 2017;36(5):1259-1265. doi:<https://doi.org/10.1016/j.clnu.2016.10.009>

21. Zeng L, Hu S, Chen P, Wei W, Tan Y. Macronutrient Intake and Risk of Crohn’s Disease: Systematic Review and Dose–Response Meta-Analysis of Epidemiological Studies. *Nutrients*. 2017;9(5):500.

22. Yang Y, Xiang L, He J. Beverage intake and risk of Crohn disease: A meta-analysis of 16 epidemiological studies. *Medicine*. May 2019;98(21):e15795. doi:10.1097/MD.0000000000015795

23. Nie JY, Zhao Q. Beverage consumption and risk of ulcerative colitis: Systematic review and meta-analysis of epidemiological studies. *Medicine*. Dec 2017;96(49):e9070. doi:10.1097/MD.0000000000009070

24. Li T, Qiu Y, Yang HS, et al. Systematic review and meta-analysis: Association of a pre-illness Western dietary pattern with the risk of developing inflammatory bowel disease. *J Dig Dis*. Jul 2020;21(7):362-371. doi:10.1111/1751-2980.12910

25. Narula N, Chang NH, Mohammad D, et al. Food Processing and Risk of Inflammatory Bowel Disease: A Systematic Review and Meta-Analysis. *Clin Gastroenterol Hepatol*. Sep 2023;21(10):2483-2495.e1. doi:10.1016/j.cgh.2023.01.012

26. Babaei A, Pourmotabbed A, Talebi S, et al. The association of ultra-processed food consumption with adult inflammatory bowel disease risk: a systematic review and dose-response meta-analysis of 4 035 694 participants. *Nutr Rev*. 2023:nuad101. doi:10.1093/nutrit/nuad101

27. Larsson SC, Burgess S. Appraising the causal role of smoking in multiple diseases: A systematic review and meta-analysis of Mendelian randomization studies. *eBioMedicine*. 2022;82doi:10.1016/j.ebiom.2022.104154

28. Piovani D, Pansieri C, Kotha SRR, et al. Ethnic Differences in the Smoking-related Risk of Inflammatory Bowel Disease: A Systematic Review and Meta-analysis. *Journal of Crohn's and Colitis*. 2021;15(10):1658-1678. doi:10.1093/ecco-jcc/jjab047

29. Jones DT, Osterman MT, Bewtra M, Lewis JD. Passive smoking and inflammatory bowel disease: a meta-analysis. *Am J Gastroenterol*. Sep 2008;103(9):2382-93. doi:10.1111/j.1572-0241.2008.01999.x

30. Mahid SS, Minor KS, Soto RE, Hornung CA, Galandiuk S. Smoking and inflammatory bowel disease: a meta-analysis. *Mayo Clin Proc*. Nov 2006;81(11):1462-71. doi:10.4065/81.11.1462

31. Labarca G, Drake L, Horta G, et al. Association between inflammatory bowel disease and chronic obstructive pulmonary disease: a systematic review and meta-analysis. *BMC Pulm Med*. 2019/10/28 2019;19(1):186. doi:10.1186/s12890-019-0963-y

32. Tiong HT, Fan D, Frampton C, Ananthakrishnan AN, Gearry RB. Physical Activity Is Associated With A Decreased Risk Of Developing Inflammatory Bowel Disease: A Systematic Review And Meta-Analysis. *J Crohns Colitis*. Apr 10 2024;doi:10.1093/ecco-jcc/jjae053

33. Wang Q, Xu K-Q, Qin X-R, Lu W, Liu Y, Wang X-Y. Association between physical activity and inflammatory bowel disease risk: A meta-analysis. *Digestive and Liver Disease*. 2016;48(12):1425-1431.

34. Chan SSM, Chen Y, Casey K, et al. Obesity is Associated With Increased Risk of Crohn&#x2019;s disease, but not Ulcerative Colitis: A Pooled Analysis of Five Prospective Cohort Studies. *Clin Gastroenterol Hepatol*. 2022;20(5):1048-1058. doi:10.1016/j.cgh.2021.06.049

35. Milajerdi A, Abbasi F, Esmaillzadeh A. A systematic review and meta-analysis of prospective studies on obesity and risk of inflammatory bowel disease. *Nutr Rev*. 2022;80(3):479-487. doi:10.1093/nutrit/nuab028

36. Rahmani J, Kord-Varkaneh H, Hekmatdoost A, et al. Body mass index and risk of inflammatory bowel disease: A systematic review and dose-response meta-analysis of cohort studies of over a million participants. *Obes Rev*. Sep 2019;20(9):1312-1320. doi:10.1111/obr.12875

37. Dong J, Chen Y, Tang Y, et al. Body Mass Index Is Associated with Inflammatory Bowel Disease: A Systematic Review and Meta-Analysis. *PLoS One*. 2015;10(12):e0144872. doi:10.1371/journal.pone.0144872

38. Wang X, Fan X, Deng H, et al. Use of oral contraceptives and risk of ulcerative colitis - A systematic review and meta-analysis. *Pharmacol Res*. Jan 2019;139:367-374. doi:10.1016/j.phrs.2018.11.036

39. Ortizo R, Lee SY, Nguyen ET, Jamal MM, Bechtold MM, Nguyen DL. Exposure to oral contraceptives increases the risk for development of inflammatory bowel disease: a meta-analysis of case-controlled and cohort studies. *Eur J Gastroenterol Hepatol*. 2017;29(9)

40. Cornish JA, Tan E, Simillis C, Clark SK, Teare J, Tekkis PP. The risk of oral contraceptives in the etiology of inflammatory bowel disease: a meta-analysis. *Am J Gastroenterol*. Sep 2008;103(9):2394-400. doi:10.1111/j.1572-0241.2008.02064.x

41. Godet PG, May GR, Sutherland LR. Meta-analysis of the role of oral contraceptive agents in inflammatory bowel disease. *Gut*. Nov 1995;37(5):668-73. doi:10.1136/gut.37.5.668

42. Yu C-L, Chou P-Y, Liang C-S, et al. Isotretinoin Exposure and Risk of Inflammatory Bowel Disease: A Systematic Review with Meta-Analysis and Trial Sequential Analysis. *Am J Clin Dermatol*. 2023/09/01 2023;24(5):721-730. doi:10.1007/s40257-023-00765-9

43. Lee SY, Jamal MM, Nguyen ET, Bechtold ML, Nguyen DL. Does exposure to isotretinoin increase the risk for the development of inflammatory bowel disease? A meta-analysis. *Eur J Gastroenterol Hepatol*. 2016;28(2)

44. Etminan M, Bird ST, Delaney JA, Bressler B, Brophy JM. Isotretinoin and Risk for Inflammatory Bowel Disease: A Nested Case-Control Study and Meta-analysis of Published and Unpublished Data. *JAMA Dermatology*. 2013;149(2):216-220. doi:10.1001/jamadermatol.2013.1344

45. Shastri SA, Kantamneni R, Rashid M, et al. Proton pump inhibitors use and risk of inflammatory bowel diseases: a meta-analysis of observational studies. *Med Pharm Rep*. Oct 2022;95(4):357-369. doi:10.15386/mpr-2259

46. Zhu JK, Ayinuer W, Gulipiye A, Aikepaer A, Hasiyeti A, Wang JL. Antibiotic Exposure and Risk of New-Onset Ulcerative Colitis: a Systematic Review and Meta-Analysis. *Clin Lab*. Oct 1 2024;70(10)doi:10.7754/Clin.Lab.2024.240312

47. Duan R, Zhang C, Li G, Li J, Duan L. Antibiotic Exposure and Risk of New-Onset Inflammatory Bowel Disease: A Systematic Review and Dose-Response Meta-Analysis. *Clin Gastroenterol Hepatol*. 2024;doi:10.1016/j.cgh.2024.02.010

48. Dar SH, Maniya MT, Merza N, et al. The association of antibiotic exposure with new-onset inflammatory bowel disease: A systematic review and meta-analysis. *Clinics and research in hepatology and gastroenterology*. May 2023;47(6):102129. doi:10.1016/j.clinre.2023.102129

49. Zou Y, Wu L, Xu W, et al. Correlation between antibiotic use in childhood and subsequent inflammatory bowel disease: a systematic review and meta-analysis. *Scand J Gastroenterol*. Mar 2020;55(3):301-311. doi:10.1080/00365521.2020.1737882

50. Ungaro R, Bernstein CN, Gearry R, et al. Antibiotics Associated With Increased Risk of New-Onset Crohn’s Disease But Not Ulcerative Colitis: A Meta-Analysis. *Official journal of the American College of Gastroenterology | ACG*. 2014;109(11)

51. Kong G, Liu Z, Lu Y, Li M, Guo H. The association between Helicobacter pylori infection and inflammatory bowel disease in children: A systematic review with meta-analysis. *Medicine*. 2023;102(36)

52. Zhong Y, Zhang Z, Lin Y, Wu L. The Relationship Between <em>Helicobacter pylori</em> and Inflammatory Bowel Disease. *Arch Iran Med*. 2021/4/1 2021;24(4):317-325. doi:10.34172/aim.2021.44

53. Shirzad-Aski H, Besharat S, Kienesberger S, et al. Association Between Helicobacter pylori Colonization and Inflammatory Bowel Disease: A Systematic Review and Meta-Analysis. *J Clin Gastroenterol*. 2021;55(5)

54. Tepler A, Narula N, Peek RM, Jr., et al. Systematic review with meta-analysis: association between Helicobacter pylori CagA seropositivity and odds of inflammatory bowel disease. *Aliment Pharmacol Ther*. Jul 2019;50(2):121-131. doi:10.1111/apt.15306

55. Wang W, Xu X. Correlation between Helicobacter pylori infection and Crohn’s disease: a meta-analysis. *Eur Rev Med Pharmacol Sci*. 2019;23(23):10509-10516. doi:10.26355/eurrev_201912_19691

56. Castano-Rodriguez N, Kaakoush NO, Lee WS, Mitchell HM. Dual role of Helicobacter and Campylobacter species in IBD: a systematic review and meta-analysis. *Gut*. Feb 2017;66(2):235-249. doi:10.1136/gutjnl-2015-310545

57. Wu XW, Ji HZ, Yang MF, Wu L, Wang FY. Helicobacter pylori infection and inflammatory bowel disease in Asians: a meta-analysis. *World journal of gastroenterology : WJG*. Apr 21 2015;21(15):4750-6. doi:10.3748/wjg.v21.i15.4750

58. Yu Q, Zhang S, Li L, et al. Enterohepatic Helicobacter Species as a Potential Causative Factor in Inflammatory Bowel Disease: A Meta-Analysis. *Medicine*. 2015;94(45)

59. Rokkas T, Gisbert JP, Niv Y, O'Morain C. The association between Helicobacter pylori infection and inflammatory bowel disease based on meta-analysis. *United European Gastroenterol J*. Dec 2015;3(6):539-50. doi:10.1177/2050640615580889

60. Luther J, Dave M, Higgins PD, Kao JY. Association between Helicobacter pylori infection and inflammatory bowel disease: a meta-analysis and systematic review of the literature. *Inflamm Bowel Dis*. Jun 2010;16(6):1077-84. doi:10.1002/ibd.21116

61. Pineton de Chambrun G, Dauchet L, Gower-Rousseau C, Cortot A, Colombel J-F, Peyrin-Biroulet L. Vaccination and Risk for Developing Inflammatory Bowel Disease: A Meta-Analysis of Case&#x2013;Control and Cohort Studies. *Clin Gastroenterol Hepatol*. 2015;13(8):1405-1415.e1. doi:10.1016/j.cgh.2015.04.179

62. Huang W, Zhang Y-d, Wang P, et al. HIV infection increases the risk of inflammatory bowel disease: a systematic review and meta-analysis. *BMC Infectious Diseases*. 2024/09/27 2024;24(1):1030. doi:10.1186/s12879-024-09964-z

63. Zhang L, Hu C, Zhang Z, et al. Association between prior appendectomy and the risk and course of Crohn's disease: A systematic review and meta-analysis. *Clinics and research in hepatology and gastroenterology*. 2023/03/01/ 2023;47(3):102090. doi:<https://doi.org/10.1016/j.clinre.2023.102090>

64. Deng P, Wu J. Meta-analysis of the association between appendiceal orifice inflammation and appendectomy and ulcerative colitis. *Rev Esp Enferm Dig*. Jul 2016;108(7):401-10. doi:10.17235/reed.2016.4176/2015

65. Kaplan GG, Jackson T, Sands BE, Frisch M, Andersson RE, Korzenik J. The risk of developing Crohn's disease after an appendectomy: a meta-analysis. *Am J Gastroenterol*. Nov 2008;103(11):2925-31. doi:10.1111/j.1572-0241.2008.02118.x

66. Koutroubakis IE, Vlachonikolis IG. Appendectomy and the development of ulcerative colitis: results of a metaanalysis of published case-control studies. *Am J Gastroenterol*. 2000/01/01/ 2000;95(1):171-176. doi:<https://doi.org/10.1016/S0002-9270(99)00739-X>

67. Kermansaravi M, Valizadeh R, Farazmand B, et al. De Novo Inflammatory Bowel Disease Following Bariatric Surgery: a Systematic Review and Meta-analysis. *Obes Surg*. Oct 2022;32(10):3426-3434. doi:10.1007/s11695-022-06226-2

68. Sun W, Han X, Wu S, Yang C. Tonsillectomy and the risk of inflammatory bowel disease: A systematic review and meta-analysis. *Journal of gastroenterology and hepatology*. Jun 2016;31(6):1085-94. doi:10.1111/jgh.13273

69. Xiong HF, Wang B, Zhao ZH, et al. Tonsillectomy and inflammatory bowel disease: a meta-analysis. *Colorectal Dis*. 2016/05/01 2016;18(5):O145-O153. doi:<https://doi.org/10.1111/codi.13323>

70. Holmes EA, Xiang F, Lucas RM. Variation in incidence of pediatric Crohn's disease in relation to latitude and ambient ultraviolet radiation: a systematic review and analysis. *Inflamm Bowel Dis*. Apr 2015;21(4):809-17. doi:10.1097/MIB.0000000000000320

71. Lu C, Yang J, Yu W, et al. Association between 25(OH)D Level, Ultraviolet Exposure, Geographical Location, and Inflammatory Bowel Disease Activity: A Systematic Review and Meta-Analysis. *PLoS One*. 2015;10(7):e0132036. doi:10.1371/journal.pone.0132036

72. Del Pinto R, Pietropaoli D, Chandar AK, Ferri C, Cominelli F. Association Between Inflammatory Bowel Disease and Vitamin D Deficiency: A Systematic Review and Meta-analysis. *Inflamm Bowel Dis*. Nov 2015;21(11):2708-17. doi:10.1097/MIB.0000000000000546

73. Wang L, Wang ZT, Hu JJ, Fan R, Zhou J, Zhong J. Polymorphisms of the vitamin D receptor gene and the risk of inflammatory bowel disease: a meta-analysis. *Genet Mol Res*. Apr 8 2014;13(2):2598-610. doi:10.4238/2014.April.8.2

74. Xue L-N, Xu K-Q, Zhang W, Wang Q, Wu J, Wang X-Y. Associations Between Vitamin D Receptor Polymorphisms and Susceptibility to Ulcerative Colitis and Crohn's Disease: A Meta-analysis. *Inflamm Bowel Dis*. 2013;19(1):54-60. doi:10.1002/ibd.22966

75. Liu C, Liu X, Shi H, et al. The correlation between serum 25-hydroxyvitamin D level and ulcerative colitis: a systematic review and meta-analysis. *Eur J Gastroenterol Hepatol*. Dec 1 2023;35(12):1375-1381. doi:10.1097/meg.0000000000002670

76. Song C, Yang J, Ye W, et al. Urban-rural environmental exposure during childhood and subsequent risk of inflammatory bowel disease: a meta-analysis. *Expert Rev Gastroenterol Hepatol*. Jun 2019;13(6):591-602. doi:10.1080/17474124.2018.1511425

77. Soon IS, Molodecky NA, Rabi DM, Ghali WA, Barkema HW, Kaplan GG. The relationship between urban environment and the inflammatory bowel diseases: a systematic review and meta-analysis. *BMC gastroenterology*. May 24 2012;12:51. doi:10.1186/1471-230X-12-51
